# Out-of-Africa migration and clonal expansion of a recombinant Epstein-Barr virus drives frequent nasopharyngeal carcinoma in southern China

**DOI:** 10.1093/nsr/nwae438

**Published:** 2024-11-28

**Authors:** Xinyi Zhang, Yanhong Chen, Jingtong Liang, Yue Yang, Hui Chen, Zehui Chen, Minhao Li, Shuanghui Chen, Tingting Chen, Haopeng He, Yunsong Liu, Zhiyuan Liu, Lu Han, Dafei Wu, Zhengting Zou, Yanhua Qu, Mingkun Li, Mark Stoneking, Qiaomei Fu, Shuhua Xu, Yi-Xin Zeng, Liang Ma, Jianjun Liu, Miao Xu, Weiwei Zhai

**Affiliations:** Key Laboratory of Zoological Systematics and Evolution, Institute of Zoology, Chinese Academy of Sciences, Beijing 100101, China; University of the Chinese Academy of Sciences, Beijing 100049, China; Human Genetics, Genome Institute of Singapore, Agency for Science, Technology and Research (A*STAR), Singapore 138672, Singapore; State Key Laboratory of Oncology in South China, Collaborative Innovation Center for Cancer Medicine, Guangdong Key Laboratory of Nasopharyngeal Carcinoma Diagnosis and Therapy, Sun Yat-sen University Cancer Center, Guangzhou 510060, China; State Key Laboratory of Oncology in South China, Collaborative Innovation Center for Cancer Medicine, Guangdong Key Laboratory of Nasopharyngeal Carcinoma Diagnosis and Therapy, Sun Yat-sen University Cancer Center, Guangzhou 510060, China; Key Laboratory of Zoological Systematics and Evolution, Institute of Zoology, Chinese Academy of Sciences, Beijing 100101, China; University of the Chinese Academy of Sciences, Beijing 100049, China; Human Genetics, Genome Institute of Singapore, Agency for Science, Technology and Research (A*STAR), Singapore 138672, Singapore; MGI Tech Singapore Pte. Ltd, Singapore 138567, Singapore; Key Laboratory of Vertebrate Evolution and Human Origins, Institute of Vertebrate Paleontology and Paleoanthropology, Chinese Academy of Sciences, Beijing 100044, China; Key Laboratory of Zoological Systematics and Evolution, Institute of Zoology, Chinese Academy of Sciences, Beijing 100101, China; Department of Biology, University of Pennsylvania, Philadelphia 19104, USA; State Key Laboratory of Genetic Engineering, Human Phenome Institute, Zhangjiang Fudan International Innovation Center, Center for Evolutionary Biology, School of Life Sciences, Fudan University, Shanghai 200433, China; Key Laboratory of Zoological Systematics and Evolution, Institute of Zoology, Chinese Academy of Sciences, Beijing 100101, China; University of the Chinese Academy of Sciences, Beijing 100049, China; Key Laboratory of Zoological Systematics and Evolution, Institute of Zoology, Chinese Academy of Sciences, Beijing 100101, China; Key Laboratory of Zoological Systematics and Evolution, Institute of Zoology, Chinese Academy of Sciences, Beijing 100101, China; Key Laboratory of Zoological Systematics and Evolution, Institute of Zoology, Chinese Academy of Sciences, Beijing 100101, China; University of the Chinese Academy of Sciences, Beijing 100049, China; Key Laboratory of Zoological Systematics and Evolution, Institute of Zoology, Chinese Academy of Sciences, Beijing 100101, China; University of the Chinese Academy of Sciences, Beijing 100049, China; Key Laboratory of Zoological Systematics and Evolution, Institute of Zoology, Chinese Academy of Sciences, Beijing 100101, China; Key Laboratory of Zoological Systematics and Evolution, Institute of Zoology, Chinese Academy of Sciences, Beijing 100101, China; Key Laboratory of Zoological Systematics and Evolution, Institute of Zoology, Chinese Academy of Sciences, Beijing 100101, China; Key Laboratory of Genomic and Precision Medicine, Beijing Institute of Genomics, Chinese Academy of Sciences, and China National Center for Bioinformation, Beijing 100101, China; Department of Evolutionary Genetics, Max Planck Institute for Evolutionary Anthropology, Leipzig 04103, Germany; Key Laboratory of Vertebrate Evolution and Human Origins, Institute of Vertebrate Paleontology and Paleoanthropology, Chinese Academy of Sciences, Beijing 100044, China; State Key Laboratory of Genetic Engineering, Human Phenome Institute, Zhangjiang Fudan International Innovation Center, Center for Evolutionary Biology, School of Life Sciences, Fudan University, Shanghai 200433, China; State Key Laboratory of Oncology in South China, Collaborative Innovation Center for Cancer Medicine, Guangdong Key Laboratory of Nasopharyngeal Carcinoma Diagnosis and Therapy, Sun Yat-sen University Cancer Center, Guangzhou 510060, China; Key Laboratory of Zoological Systematics and Evolution, Institute of Zoology, Chinese Academy of Sciences, Beijing 100101, China; Human Genetics, Genome Institute of Singapore, Agency for Science, Technology and Research (A*STAR), Singapore 138672, Singapore; State Key Laboratory of Oncology in South China, Collaborative Innovation Center for Cancer Medicine, Guangdong Key Laboratory of Nasopharyngeal Carcinoma Diagnosis and Therapy, Sun Yat-sen University Cancer Center, Guangzhou 510060, China; Key Laboratory of Zoological Systematics and Evolution, Institute of Zoology, Chinese Academy of Sciences, Beijing 100101, China; Center for Excellence in Animal Evolution and Genetics, Chinese Academy of Sciences, Kunming 650223, China

**Keywords:** Epstein-Barr virus, Nasopharyngeal carcinoma, out-of-Africa migration, co-evolution, adaptation, recombination

## Abstract

While Epstein-Barr virus (EBV) infection is ubiquitous globally, a high-risk EBV subtype associated with the extremely high incidence of nasopharyngeal carcinoma (NPC) was found in southern China, but the evolution history of EBV in humans and the origin of this high-risk subtype remains enigmatic. By obtaining one of the largest datasets of EBV genomes across the world, we found that EBV had an evolutionary history matching the out-of-Africa migration of humans. Within the high-risk subtype from southern China, we identified a rapidly expanding clonal strain originating from a recombination event between EBV strains from northern and southern Chinese around 4000 years ago, followed by strong Darwinian evolution with a fitness advantage of 4%. The clonal strain has almost doubled the risk for NPC compared to the high-risk subtype and explained around 66% of the NPCs, representing the highest risk factor for NPC identified so far. Taken together, we unraveled a strong co-evolution history between EBV and humans where human migration and admixture triggered subsequent recombination and expansion of a highly advantageous EBV strain, leading to a cancer epidemic in southern China.

## INTRODUCTION

Epstein-Barr virus (EBV) is the first identified oncogenic virus, persistently infecting >90% of the human populations. Despite its frequent asymptomatic persistence after primary infection, EBV has been linked to a wide spectrum of malignancies, including endemic Burkitt's lymphoma (BL), Hodgkin lymphoma (HL), NK/T cell lymphomas, a subtype of gastric cancer (GC), nasopharyngeal cancer (NPC) [1] as well as recently reported multiple sclerosis (MS) [2,3]. One remarkable observation is that many of these EBV-associated diseases have distinctive geographic distributions and ethnic disparities [4]. For instance, BL is prevalent predominantly in parts of Africa with high malaria incidence, while MS is most frequent in northern Europe, but very rare in Asians [5]. Among EBV-related diseases, NPC has the most significant population disparity, with a more than 20-fold increase in incidence rates in southern China (13.9–25.0/100 000 per year, aka Cantonese Cancer) [6,7]. How a common virus can contribute to endemic diseases in geographically different populations is poorly understood.

Previous EBV genomic studies have revealed a strong population structure correlated with ethno-geographic regions [8,9], holding the clues to addressing population disparities in EBV-related diseases. Even though EBV variations have been shown to contribute to a number of diseases including lymphomagenesis [10] and MS [11], one of the most compelling associations linking EBV subtypes to locally endemic diseases comes from NPC [9,12–14]. Even though genetic associations in human populations identified a number of risk variants including *HLA* and *CDKN2A* [15,16], the contribution of these loci to the overall risk seems to be rather modest. Recent studies have revealed that a high-risk EBV subtype remarkably prevalent among the southern Chinese population, was the most significant risk factor identified to date with an estimated odds ratio (OR) as large as 7.6 for NPC and contributed over 80% of the population risk of NPC in southern China [9,12–14]. However, the origin of the high-risk strain and the reasons for its endemic prevalence remain elusive.

As a member of the primate-specific gamma-1 herpesvirus, which belongs to genus *Lymphocryptovirus*, EBV has been co-evolving with us since human origin [17]. Most individuals are asymptomatic carriers of EBV, maintaining an exquisite balance between host and pathogen. NPC, like many other EBV-related malignancies, represents a rare consequence of EBV infection. The emergence of NPC in southern China posits a puzzling scenario, which has been hypothesized to be related to the transmission of genetic risk factors or life history traits (e.g. salt fish consumption) to the Han Chinese from ancient Bai-Yue (Tai-Kadai speaking people living in southern China) through the admixture of these two ethnic groups [18]. How EBV could have evolved with us since our migration out of Africa and how the high-risk strain has arisen, driving the origin of NPC in southern China remains unknown.

In this study, we sequenced and assembled one of the largest datasets of EBV genome sequences from healthy controls and individuals with EBV-related diseases. Through population genetic analyses, we dissected the global distribution of EBV and explored the relationship between humans and EBVs. Interestingly, among the high-risk subtype for NPC, we discovered a rapidly expanding clonal strain which has doubled the risk for NPC compared to the high-risk subtype, representing the highest risk for NPC identified so far. Through molecular genetic analysis, we dissected the evolutionary origin of the clonal strain and explored how Darwinian evolution might drive the rapid rise of this strain. Taken together, we unraveled an interesting case of how human migration had triggered a highly-pathogenic recombinant pathogen followed by a subsequent clonal expansion, leading to a cancer epidemic in southern China.

## RESULTS

### Sequencing and curation a global dataset of EBV genomes

**Figure 1.**
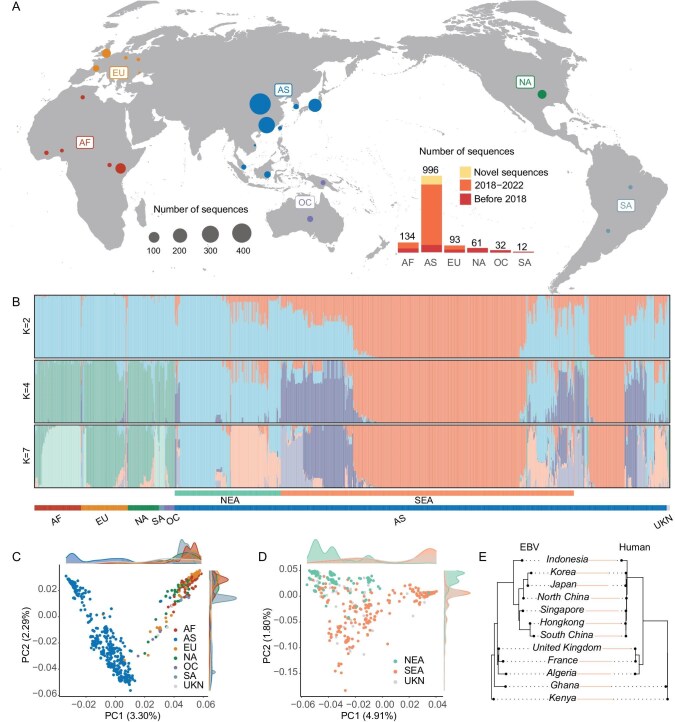
Population structure and history of EBV. (A) Distribution of EBV strains across the world. The inset plot displays the sample-size distribution across time and geography. Sequences with unknown geographic origin were omitted from the plot. (B) Population structure analysis of all the type 1 strains (*n* = 1205) with varying number of population groupings (K = 2,4, or 7). Strains were labeled with their geographic origin. AF: Africa, EU: Europe, NA: North America, SA: South America, OC: Oceania, AS: Asia, UKN: Unknown geography, NEA: North East Asia, SEA: South East Asia. (C) Principal component analysis (PCA) of the type 1 EBV strains. The first two components explaining 3.30% and 2.29% of the total variance, respectively. Strains were labeled according to their geographic origin. (D) Principal component analysis (PCA) of the type 1 strains from East Asia. Strains from northern and southern China were labelled in different colors. (E) Maximum likelihood tree of the EBV populations (left) and humans (right) constructed using TreeMix. The two trees had minor differences in topology with a Robinson-Fould distance of 2. Review drawing number: GS 京 (2025) 0056号.

Even though most humans carry EBV, due to the trace quantity of EBV genomes in human samples, large scale sequencing of EBV genomes from major human populations has only recently gained momentum after the employment of capture sequencing technology [8,19]. In order to understand the prevalence and distribution of the previously identified high-risk EBV subtype, we performed capture sequencing of EBV genomes from 118 individuals with multiple diseases including NPC (*n* = 68), GC (*n* = 25), nasopharyngitis (*n* = 6) and Natural killer/T-cell lymphoma (NKTL, *n* = 19) across northern and southern China. To better understand EBV's global distribution and evolution, we retrieved all available EBV genomic sequences (*n* = 1360) from the NCBI database covering all major continental populations (Fig. 1A, Supplementary data). Indeed, 78% of all sequences were generated after 2018 with a strong representation of strains from East Asia (Fig. 1A inset). After quality control and multiple sequences alignment (Figs S1 and S2), we curated one of the largest EBV dataset (*n* = 1334) covering all major continental areas with strains from Africa (*n* = 134), Asia (*n* = 996), Europe (*n* = 93), North America (*n* = 61), South America (*n* = 12), Oceania (*n* = 32) and unknown origin (*n* = 6) (denoted as dataset 1, Fig. 1A, Tables S1 and S2). Across the dataset, EBV sequences were collected from both healthy (∼20%) as well as diseased individuals (e.g. different cancer types, Fig. S3).

### The evolutionary history of EBV genomes mirrors recent human migration

Even though the population structure of EBV genomes across the world has been explored in several previous studies with smaller numbers of sequences [20–22], our large collection of EBV genomes not only depicts the global distribution, but also allow us to dissect finer-scale population substructures in EBV genomes within each continent. When we performed a principal component analysis (PCA) of all 1334 sequences, we discovered two major groups of EBV strains driven by strong differentiation in the *EBNA2* and *EBNA3* genes, representing type 1 and type 2 EBVs (Figs S4 and S5) [8,23]. Focusing on the type 1 EBV which is a globally distributed subtype (*n* = 1205, denoted as dataset 2), we found that strains from Europe and Africa are less differentiated from each other and sequences from Asia are genetically more dissimilar from the rest of the world (K = 2 in Fig. 1B and 1C, Fig. S6) [8,21]. With increasing number of clusters in the admixture analysis [24], we observed serial partitioning of geographic subpopulations including the separation between the northern and southern Asian populations (K = 4, Fig. 1B) as well as between major continental populations (e.g. K = 7 for Africans, Fig. 1B, D, Fig. S6). As the current dataset has a significant proportion of Asian strains which can influence the structure analysis, we created a balanced subset of EBV genomes by subsampling similar numbers of sequences from each continent. The balanced dataset not only allowed us to confirm both broad differences between continental populations, but also fine-scale differentiation within subcontinents (e.g. northern and southern China; Fig. S7). When we further extended the analysis to healthy samples only, we observed similar population structure among continental EBV populations (Fig. S8).

Even though the strong population structure in EBV genomes associated with ethno-geographic ranges was known, the high concordance between geographic location and genetic differentiation raised an interesting question: what is the evolutionary relationship between continental subpopulations of EBV? Previous studies have often focused on the phylogenetic relationship between all the EBV genomes which can be obfuscated by gene flow between populations or recombination. However, the global collection of EBV genomes allow us to infer the population relationship (i.e. population branching history) between EBV populations based on the joint allele frequencies of variants across the genome. By selecting geographic regions with sufficient numbers of EBV sequences (*n* ≥ 5 for each region), we inferred a population history of EBV populations. Interestingly, the EBV population history had a topological relationship largely matching human history (i.e. (Africa, (Europe, Asia))), where lineages from Africa (e.g. Kenya) branch off first from the basal position, followed by the divergence of two sister clades from Europe and Asia, respectively. Therefore, the overall branching order of EBV populations seems to largely mimic the history of humans. The long branch leading to the Asian populations matches the high differentiation of Asian EBVs from the rest of the world in the PCA (Fig. 1C), possibly driven by historical bottlenecks along the Asian lineage. To further explore the fine-scale correlation between the human and viral history, we inferred the evolutionary history of the corresponding human populations (see Methods, Table S3, Fig. S9) and found that human history matches almost perfectly with the history of EBV with a very minor topological difference (i.e. Robinson-Foulds distance =2, Fig. 1E). Thus, the high concordance strongly suggests a co-evolution history between EBVs and humans (see later sections).

### A clonal strain with much higher NPC risk was found in southern China

**Figure 2.**
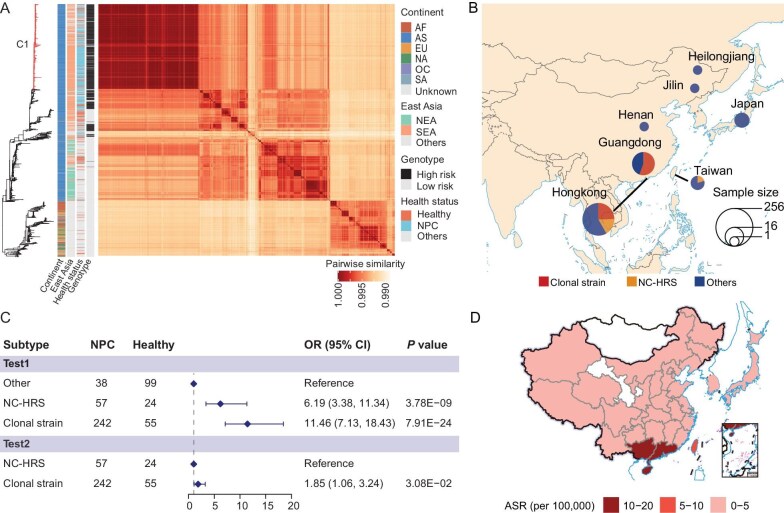
The geographic distribution and the risk association of clonal strain. (A) Reconstructed phylogenetic tree from Gubbins after filtering putative recombinant regions (see Methods). A heatmap representing the pairwise similarity between all strains is shown. A group of highly similar sequences (denoted as the clonal clade) is identified (in red block). High risk strain is defined as strains carrying C-C-T alleles at position 162215, 162476 and 163364. (B) Frequencies of the clonal strain in healthy individuals across East Asia. (C) The odds ratio of NPC risk for the clonal, NC-HRS as well as other strains in the population. (D) The geographic distribution of NPC incidence (age-standardized incidence rate or ASR) across East Asia (see Methods). Review drawing number: GS 京 (2025) 0056号.

Recent genome-wide association studies (GWAS) of EBV genomes discovered a group of high-risk EBV haplotypes strongly associated with the elevated NPC incidence in southern China [9,12–14] (denoted as the southern China high-risk strain for NPC, abbreviated as high-risk subtype or HRS, Fig. S10). Interestingly, among the high-risk subtype, a clonal expansion was initially discovered [9], but its distribution and evolutionary origin remain unknown. In order to understand the prevalence and geographic distribution of this clonal strain, we inferred the evolutionary relationship between all the unique type 1 EBV genomes (*n* = 1192, unique sequences from dataset 2, see Methods) taking into account ancestral recombination (see Methods, Figs S11 and S12). Indeed, we found a group of highly similar EBV genomes within the high-risk subtype in southern China (denoted as the clonal strain or C1, Fig. 2A). Other strains outside of the clonal clade within the high-risk subtype were named as the non-clonal high-risk subtype (NC-HRS). We found that the clonal strains accounted for a significant proportion (i.e. 55.6% in Guangdong and 24.6% in Hong Kong) of healthy individuals, but are rare in other geographic locations (Fig. 2B, Table S4), suggesting a localized expansion of the clonal strain within southern China (Fig. 2B).

Looking across all the sequences, we found that NPC cases from endemic southern China (including Guangdong, Guangxi and Hong Kong) were highly enriched for the clonal strain (Fig. 2A). As our previous GWAS study found that the high-risk subtype has a much-increased NPC risk with an odds ratio (OR) of 7.6 [9], when we compared the risk effect of the clonal strain and the NC-HRS, we found that the NPC risk effect for the clonal strain was significantly larger (OR = 11.46; 95% CI = 7.13–18.43) than the NC-HRS (OR = 6.19, 95% CI = 3.38–11.34, Fig. 2C, Table S5) when using other EBV subtypes (i.e. non high-risk subtype) from southern China as the baseline reference. The clonal strain had almost doubled the NPC risk compared to the NC-HRS (OR = 1.85, CI = 1.06–3.24, Fig. 2C, Table S5), an effect size comparable to monogenic high penetrance mutations. Therefore, the NPC risk associated with the clonal strain was far greater than any other known risk factors for NPC, leading to a strong match between the EBV distribution (Fig. 2B) and NPC incidence (Fig. 2D).

### A north-south recombination event drives the origin of the clonal strain

**Figure 3.**
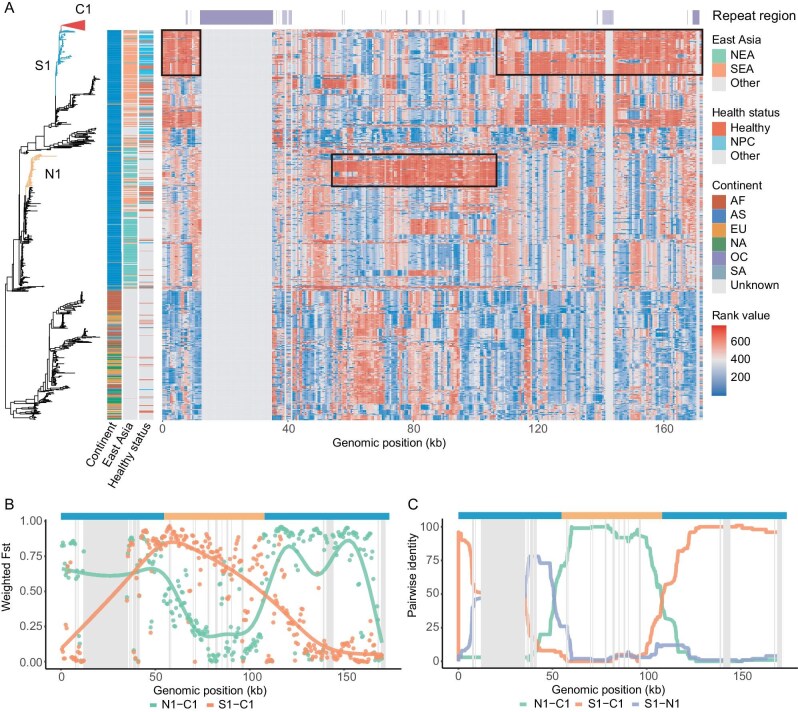
The recombination history of the clonal strain. (A) Sequence comparison between the clonal clade and all the other sequences. The phylogenetic relationship on the left is the same as presented in Fig. 2A, but with the clonal clade collapsed as a red triangle. Three distinctive clades (clonal clade C1, subgroup of southern strains S1 and a subgroup of northern strains N1) are labelled on the phylogenetic tree. Heatmap of rank values of the sequence similarity to the clonal strain are plotted. Regions with high similarity (i.e. high rank values) are boxed in black rectangles. Repeat regions were marked on the top panel. (B) Population differentiation (i.e. Fst) between the clonal clade (C1) and other subgroups (i.e. N1 and S1). The horizontal bar on top of the panel is the putative recombination track inferred from RDP with the blue color representing the S1 ancestry and orange color representing the N1 ancestry. (C) Output from RDP. The y-axis is the pairwise identity between three consensus sequences derived from C1, N1 and S1, while the x-axis is the coordinate of the genome. The same horizontal bar was plotted as in panel B.

The highly similar sequences together with the restricted geographic distribution for the clonal strain imply a recent origin in southern China. Moreover, similar to several previous studies [22,25], we detected extensive recombination events throughout the history of EBV sequences with the clonal strains have nearly identical recombination profiles, suggesting a possible mosaic ancestry for the clonal strains (Fig. S11). In order to systematically explore possible ancestral relationships between the clonal clade and other lineages, we first computed pairwise sequence similarity between the clonal clade and all the other sequences using a sliding-window approach (see Methods). Interestingly, we found the genomes of the clonal strain (i.e. C1) are highly similar to a subgroup of strains from southern China (i.e. denoted as S1) except the middle part of the genome (starting from ∼54k to ∼107k, spanning *EBNA3A-C* and *BZLF1*) where it is most similar to a subgroup of EBV genomes from northern China (denoted as N1). To further test the mosaic origin across the genome, we calculated genetic distance (i.e. FST [26]) between the clonal strains (i.e. C1) and the other two subgroups (i.e. S1 from southern China and N1 from northern China) and observed a strong discontinuity in the middle part of the genome where C1 is more similar to S1 on both ends of the genome, but highly similar to N1 in the middle part of the genome (Fig. 3B). Taken together, both the sequence similarity (Fig. 3A) and genetic differentiation analyses (i.e. FST, Fig. 3B) supported a mosaic ancestry for the clonal clade.

In order to formally test for the presence of recombination, we used RDP5 [27], a computational package which includes seven different methods for detecting recombination (RDP, GENECONV, BootScan, MaxChi, Chimera, SiScan and 3Seq) and tested for possible recombination events between C1, S1 and N1 (see Methods). Interestingly, all the methods implemented in RDP5 unanimously detected strong signals of this recombination event (Fig. 3C for the RDP method, *P*-value ≤ 10^−6^ across all the other methods, Table S6). Collectively, all our analyses supported that recombination between a subgroup of southern and northern strains created the highly pathogenic clonal strain for NPC.

### Molecular dating revealed a recent origin of the clonal clade

The recombination between the southern and northern strains posits an interesting question: when did the northern and southern strains come together to ‘reproduce’ the highly pathogenic clonal strain. In order to date the origin of the clonal strain, we first identified putative non-recombining genomic regions and subsequently performed molecular dating of the clonal strain based on tip dates (see Methods) [28]. Even though there are a substantial number of EBV sequences in the database, the collection dates of these sequences only span around 40 years. The short time-span of the tip branches thus yields a non-significant temporal signal in the time-calibrated phylogeny when we performed a date-randomization test (Fig. S13). One powerful alternative in molecular dating is sampling ancient specimens. However, a literature search only found a shallowly sequenced EBV genome from chewed birch pitch 5700 years ago [29]. Even though there is an appreciable divergence between the ancient and extant samples, the number of high-confidence sites from the ancient EBV genome was very limited (nt = 3775 bp) and the tip date analysis also indicated non-significant temporal information in the time-calibrated phylogeny (Fig. S13, Table S7).

**Figure 4.**
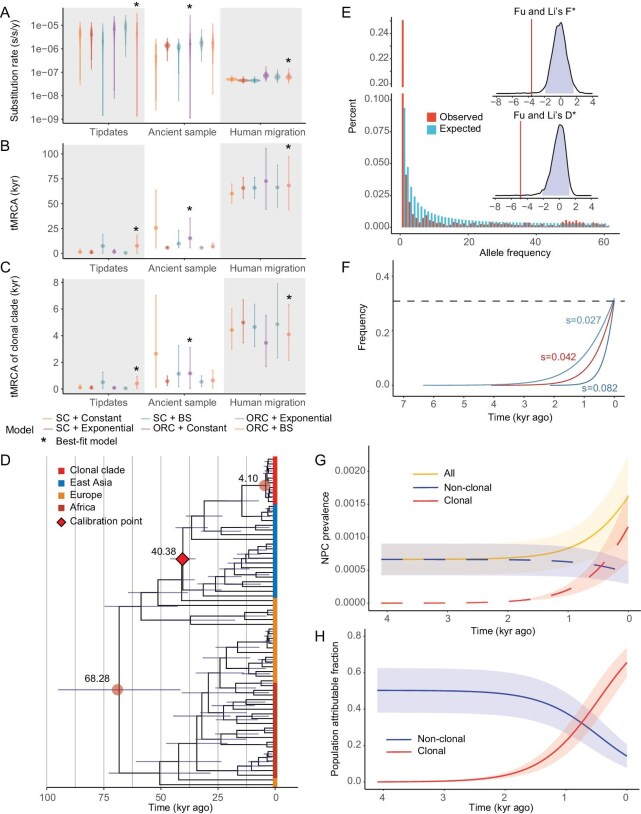
The timing of the clonal clade. (A) The substitution rate estimates (s/s/y) under different parameter settings. The models are specified as ‘clock model’_‘population prior’ where clock models can be strict clock (SC) or optimized relaxed clock (ORC) and population priors can be constant, exponential or Bayesian skyline (BS). The best-fit models are marked as *. (B) tMRCA (kyr) of all the type 1 EBV sequences under different parameter settings. (C) tMRCA of the clonal clade under different parameter settings. (D) The dated phylogeny with major nodes as well as calibration point labelled. (E) The folded site frequency spectrum of the EBV genomes in Hong Kong. The expected and observed values were plotted as discrete counts. The inset figures show the neutral distribution of Fu and Li's D* and F* values (95% CI are shaded in purple) and the observed values (as red vertical lines). (F) Frequency trajectories of the recombinant strain (i.e. C1) given the estimated time of origin (4095, CI = [2133, 6344]) and haploid selection model. The estimated selective coefficient was labelled with nearby different trajectories. (G) The prevalence of NPC cases as a function of the frequency of the clonal clade through time (in yellow, see Methods). The blue and red dashed lines represent the prevalence of NPC attributable to the clonal (red) and non-clonal strains (blue). (H) The population attributable fraction (PAF) of NPC due to the clonal strain (in red) and non-clonal strains (in blue) (see Methods).

Without enough temporal signal from tip dates, we calibrated the EBV phylogeny based on the history of human migration as we observed a broad concordance between human and EBV history (Fig. 1E). In particular, when we set the split time between Asians and Europeans to be 40.6 thousand years with a 95% confidence interval spanning 36.0 and 45.2 kyrs [30], the tMRCA of all type 1 EBVs was found to be 68 282 [43 782, 97 302] years before present in the best fitted model (Fig. 4D, Table S7), broadly matching the time of human migration out of Africa [30].

Based on this calibration, we estimated that the clonal clade arose 4095 [2133, 6344] years ago (Fig. 4D). This time of origin, i.e. the time of the recombination event between N1 and S1 strains, coincides with the estimates from ancient DNA evidence that a major episode of increased northern Chinese ancestry spreading to southern China after the Neolithic ∼3000–5000 years ago [31]. Interestingly, the estimated mutation rate by this approach (6.4$\times$10^−8^) is quite similar to the mutation rate estimates for herpes viruses (e.g. HSV-1, 3.5$\times$10^−8^ substitutions per site per year [32,33]). These estimates are quite robust when we performed the same analysis based on different subsets of datasets (Fig. S14). Taken together, even though we only calibrated part of EBV history (Asian strains vs the rest) against the divergence between Europeans and Asians, we revealed a highly concordant evolutionary history between EBVs and humans across all time.

### Strong Darwinian selection drives rapid expansion of the clonal clade and the rise of NPC in southern China

The recent origin together with the high frequency of the clonal strain in southern China suggests that Darwinian selection might be driving the rapid expansion of the clonal clade. To test this hypothesis, we plotted the folded site frequency spectra (SFS) of the variants across the EBV genomes in southern China (e.g. Hong Kong in Fig. 4E, Guangdong in Fig. S15) and observed an enrichment of both rare and intermediate frequency variants, a typical signature of ongoing Darwinian selection. Using population genetic methods targeting ongoing selective sweeps (i.e. incomplete sweep) [33], we found that both Fu and Li's D* and F* strongly deviate from the neutral expectation (Fig. 4E inset, *P*-value < 0.01 for Hong Kong), suggesting that strong adaptive evolution may have driven the rapid expansion of the clonal strain (Fig. S15).

The recent origin and highly restricted geographic distribution together with a strong local selective signal suggest that an extremely strong adaptation occurred in southern China. In order to estimate the selective advantage of the clonal strain as compared to other EBV strains from southern China, we modelled the haplotype frequency of EBVs in humans over time using the Wright-Fisher model (haploid selection model, see Methods). In order to reach an observed allele frequency of 0.309 (average frequency of the clonal strain in Guangdong and Hong Kong) in such a short period of time (i.e. 4095 years ago, 95% CI = 2133–6344 years), the fitness advantage for the clonal clade compared to other non-clonal strains was estimated to be 4.2% (95% CI = 2.7%–8.2%, Fig. 4F). The estimated selective coefficient is exceptionally strong when compared to known adaptations in model and non-model organisms [34,35]. As a corollary to the rapid rise of the clonal clade, NPC prevalence could rapidly increase from 0.069% to the current 0.163% in southern China over the past 2000 years (Fig. 4G) and the NPC risk attributable to the clonal clade could rise remarkedly from 3.68% to 65.55% (Fig. 4H, Table S8), whereas the NC-HR strains only contributed to ∼14.18% of the NPC cases. This underscores the substantial impact of the clonal strain in driving NPC risk in southern China. Taken together, the star-shaped genealogy (Fig. 2A) and recent origin (Fig. 4D) together with high regional frequency (Fig. 2B) strongly pointed to Darwinian selection as the driving force for the rapid expansion of the clonal clade, leading to the rise of NPC in southern China.

## DISCUSSION

Through sequencing and assembling one of the largest datasets of EBV genomes from all over the world, we systematically explored the population structure of EBV and subsequently found that EBV had an evolutionary history broadly matching the out-of-Africa migration of humans, suggesting a co-evolutionary history between EBVs and humans. Through recombination analysis, we found that a rapidly expanding clonal strain in southern China originated from a recombination event between EBV strains from northern and southern China. The clonal strain has almost doubled the risk for NPC compared to the previously identified high-risk EBV subtype and explains ∼66% of the NPC cases, representing the highest risk factor for NPC identified so far. Through molecular genetic analysis, we found that the clonal strain originated in southern China around 4000 years ago via a recombination event between northern and southern EBV strains. We estimated that the clonal strain then increased rapidly in frequency, driven by strong Darwinian evolution with a selective advantage as high as 4.2%. Taken together, we revealed an extraordinary journey EBV has traveled with us where adaptation initiated first by human migration and subsequently a recombination event in a tumor virus, led to a cancer epidemic in southern China.

Despite the general intuition that slowly evolving pathogens like herpesviruses are likely to co-evolve with humans [17,36], the co-evolutionary history between EBV and humans has never been discovered before. The strong latency program in B cells developed by EBV and a tendency of vertical transmission in humans [37–39] might be important mechanistic factors driving this co-evolution relationship. To our knowledge, EBV might be one of the best examples (the other examples being TB [40] and *H. Pylori* [41]) of co-evolution between humans and pathogens. The rise of the clonal strain in southern China also explains why NPC prevalence remains almost unchanged among the southern Chinese populations, despite declines in exposure to known environmental factors like smoking and salted-fish consumption over the past three decades [6].

The common hypothesis for the origin of the Cantonese cancer is that NPC was transmitted to Han Chinese from ancient Bai-Yue through admixture between these two ethnic groups [18]. The origin of the clonal strain brought together multiple pieces of evidence from human evolution and provided a new perspective on the rise of NPC in southern China. After the ancient Bai-Yue diverged from other Chinese populations 4000 to 7400 years ago [42], gene flow from northeast to southeast Asia significantly increased starting ∼3000 to 5000 years ago [31]. During this time, the most likely origin of the Cantonese cancer could be that a subgroup of northern Chinese carrying the N1 EBV lineage migrated to southern China and admixed with a subgroup of Bai-Yue people carrying the S1 EBV lineage. The population admixture allowed the N1 and S1 EBV lineages to recombine and generate the clonal strain (C1) ∼4000 years ago. The high fitness of the clonal strain together with its much-elevated NPC risk led to a rapid clonal expansion and high incidence of NPC in southern Chinese. Thus, this study discovered how human migration, population admixture, recombination, and natural selection act in concert to drive a cancer epidemic in southern China. Moreover, our findings also provide important insights into an emerging landscape where local adaptations of EBV in different human populations could lead to diverse pathological conditions.

There are a few limitations to the current study worth discussing here. First of all, as EBV associated diseases are quite distinctive in different geographic locations [8], current samples in the public database are biased due to the unique disease epidemiology (Supplementary Note 1, Figs S8 and S16). Moreover, very few healthy samples were collected except Europe and Asia, which prevent a ‘disease-free’ analysis of EBV evolution (Table S2). Even though we found that the sampling bias can often be alleviated and will not affect many of the evolutionary inferences (Supplementary Note 1), depicting a complete picture of EBV evolution across the world would require a comprehensive dataset with more balanced samples in the coming future. Second, the history of the recombination event is only partially inferred. For example, the sequence alignment to the right of the large repetitive region is quite suboptimal (Fig. S17), which prevents identifying the definitive parental source in the associated genomic region. Future studies with long read sequencing might be able to resolve this problem more thoroughly. Third, the exact mechanism of selection on the clonal strain is not yet known. Even though the higher fitness advantage of the clonal clade is correlated with increased NPC risk, it is hard to imagine natural selection will directly select on the oncogenic potential of EBV, as cancer usually occurs post-reproduction and hence would not be expected to be subject to strong selection. At this moment, it is still quite challenging to pinpoint the exact gene or mutation for the selective advantage. However, there are a few interesting observations from the literature worth discussing here. For instance, the clonal strain is found to carry a high-frequency mutation, creating a novel epitope (from IVTDFSVIK to IVTDFSVIKN) in the *EBNA-3B* gene, which was found to abolish the presentation by HLA-A*11 allele in several previous studies [43–45]. Thus, this mutation may confer a selective advantage in the southern Chinese population, where the A∗11 allele is frequent. In addition, the clonal strain may also have distinct functional properties that enhance its spread. For example, M81, a clonal strain derived from the NPC tumor, displays epitheliotropism and a high level of spontaneous replication in B cells [46]. These derived traits may facilitate its transmission, potentially irrespective of immunogenic differences between human populations. Last, it remains unknown why the clonal strain is thus far geographically constrained in southern China given the rate of migration among human populations. The short time span and biased gene flow (north-south migration) might contribute to the unique geographic range. In addition, we hypothesize that viral and human interactions could also contribute to the high fitness of the clonal strain in southern Chinese. Sequencing additional human populations together with their EBV genomes might be able to unravel a more detailed history of co-evolution between EBVs and humans in East Asia, opening further avenues for controlling and eradicating NPC in southern China.

## MATERIALS AND METHODS

For detailed materials and methods, please see the Supplementary information.

### Whole-genome sequencing of EBV genomes from China

A total of 118 tissue samples including saliva and tumor specimens were collected from China (Table S1) and sequenced using next-generation sequencing. Data preprocessing and variant identification were performed. Consensus genome sequences were obtained for each sample using a custom python script.

### Public data curation and multiple sequence alignment

We retrieved 1360 public EBV genomes from the NCBI nucleotide database (up to July, 2022) (Table S2). After multiple sequences alignment and quality control for merged public and private datasets, 1334 EBV genome sequences were retained for further analysis (Fig. S2). Single nucleotide variations (SNVs) for the aligned data were extracted by custom python script.

### Principal component analysis and population structure analysis

Principal component analysis (PCA) and PC loadings were calculated using smartpca from EIGENSOFT. Population structure analysis was performed using Admixture.

### Population history across humans and EBVs

We inferred both the EBV and human population branching history based on the joint allele frequencies across regional populations using TreeMix, we rooted both trees with the African populations and compared the trees in R.

### Recombination analysis and identification of the clonal strain

Gubbins was used for inferring recombination events and pairwise similarity was calculated in R. Here, we defined clonal strain as a group of highly similar and non-recombinant strains forming a monophyletic clade in the phylogenetic tree inferred from Gubbins. Mean similarity between genomes from the clonal clade C1 to others was calculated in R. RDP5 was used to detect the recombination events based on consensus genomes of focal clades (S1, N1, C1). Fst between samples from these clades (N1, S1 and C1) were calculated using vcftools.

### Association analysis

The association between NPC and EBV subtypes was analyzed using a logistic regression model in a cohort from Guangdong or Hong Kong (Test 1 in Fig. 2C). The logistic regression model was exclusively applied to NC-HRS and the clonal strain (Test 2 in Fig. 2C) to estimate the NPC risk associated with clonal strain compared to NC-HRS subtype. The age-standardized incidence rate (ASR, per 100 000) data were collected from public report.

### Molecular dating

Molecular dating and date-randomization tests (DRTs) were performed based on random sampling of EBV genomes. We used different priors for molecular dating. The EBV genome of a 5700 years-old sample [29] was also downloaded for molecular dating.

### Selection analysis

The actual folded site frequency spectrum (SFS) was calculated based on real data, while the expected SFS is computed under the neutral expectation. The Fu and Li's test was also performed. We then employed a haploid selection model from Population Genetics to estimate the selective coefficient of the recombinant virus.

### Contribution of different EBV subtypes to the NPC risk

The contribution of EBV strains to the NPC risk was evaluated by population attributable fraction (PAF) and NPC prevalence in response to the estimated frequency trajectory of the clonal strain over time.

## Supplementary Material

nwae438_Supplemental_Files

## Data Availability

The raw sequence data of EBV genomes reported in this paper have been deposited in the Genome Sequence Archive [47] in National Genomics Data Center [48], China National Center for Bioinformation/Beijing Institute of Genomics, Chinese Academy of Sciences (GSA: CRA015697) that are publicly accessible at https://ngdc.cncb.ac.cn/gsa.
