# Supplementary material for: Out-of-Africa migration and clonal expansion of a recombinant Epstein-Barr virus drives frequent nasopharyngeal carcinoma in southern China
Source: Natl Sci Rev. 2024 Nov 28;12(4):nwae438. doi: 10.1093/nsr/nwae438 (PMC11954593; doi:10.1093/nsr/nwae438)
Supplement: nwae438_Supplemental_Files [file nwae438_supplemental_files.zip › Supplementary Data.docx]

**Supporting Information**

**Out-of-Africa migration and clonal expansion of a recombinant Epstein-Barr virus drives frequent nasopharyngeal carcinoma in southern China**

Xinyi Zhang^1,3,4,*^, Yanhong Chen^2,*^, Jingtong Liang^2,*^, Yue Yang^1,3^, Hui Chen^4,5^, Zehui Chen^6^, Minhao Li^1,7^, Shuanghui Chen^8^, Tingting Chen^1,3^, Haopeng He^1^, Yunsong Liu^1^, Zhiyuan Liu^1,3^, Lu Han^1,3^, Dafei Wu^1^, Zhengting Zou^1^, Yanhua Qu^1^, Mingkun Li^9^, Mark Stoneking^10^, Qiaomei Fu^6^, Shuhua Xu^8^, Yi-Xin Zeng^2^, Liang Ma^1,#^, Jianjun Liu^4,#^, Miao Xu^2,#^, Weiwei Zhai^1,11,#^

*these authors contributed equally

# Correspondence should be addressed to: [maliang@ioz.ac.cn](mailto:maliang@ioz.ac.cn), [liuj3@gis.a-star.edu.sg](mailto:liuj3@gis.a-star.edu.sg), [xumiao@sysucc.org.cn](mailto:xumiao@sysucc.org.cn),[weiweizhai@ioz.ac.cn](mailto:weiweizhai@ioz.ac.cn)

**Table of content:**

**Materials and Methods**

**Supplementary Notes 1**

**Supplementary Figures** 1 to 17

**MATERIALS AND METHODS**

**Whole-genome sequencing of EBV genomes from China**

A total of 118 tissue samples including both saliva and tumor specimens were collected from three medical institutions in China: Sun Yat-sen University Cancer Center in Guangdong Province (SYSUCC), the Affiliated Hospital of Qingdao University in Shandong Province, and Ruijin Hospital in Shanghai. Details of the samples were provided in Supplementary Table 1. This study is approved by the institutional ethics committees and written informed consent was obtained from each participant before any study-related procedures were undertaken (Approval number: GZR2014-059). Saliva samples were first collected and preserved in vials containing a lysis buffer comprising 50mM Tris (pH 8.0), 50mM EDTA, 50mM sucrose, 100mM NaCl, and 1% SDS. Tumor specimens were derived from biopsy samples obtained during surgical operations and verified by histopathological examination. Both saliva and tumor samples were stored at -80 °C. DNA extraction from saliva samples was performed using the Chemagic STAR workstation by Hamilton Robotics, while tumor biopsy samples were processed with a DNeasy Blood and Tissue Kit from Qiagen. The capture of EBV genomes was accomplished using the MyGenostics GenCap Target Enrichment Protocol (GenCap Enrichment, MyGenostics). Subsequently, DNA libraries were prepared and sequenced on the Illumina HiSeq 2000 platform following standard protocols from Illumina.

Adapters and low-quality reads were trimmed off using Trim Galore (version 0.4.5)[1]. Paired-end reads were subsequently aligned to the EBV reference genome (NC_007605.1) using BWA-MEM (Burrows–Wheeler Aligner version 0.7.17)[2]. SAMtools (v1.9) as well as BCFtools (v1.9)[3] were employed for indexing, sorting, marking duplicates and filtering out low-quality reads. Following data preprocessing, single nucleotide variations (SNVs), insertions and deletions (indels) were identified using HaplotypeCaller[4] from the Genome Analysis Toolkit (GATK v4.1.0.0) and further filtered through VQSR by using high quality variants set (GQ>40) from SAMtools as the training SNP set[5].

Consensus genome sequences (fasta format) were obtained for each sample using a custom python script based on the detected variants. The average sequencing depth of 118 samples was found to be 2262×. We filtered away samples with average depth < 30 and 113 genomes who covered more than 95% length of EBV reference genome were kept for the final analysis (Supplementary Table 1 and Supplementary Fig.1).

**Public data curation and multiple sequence alignment**

By searching for EBV genomes (taxon id 10376), we successfully retrieved 1360 public EBV genomes (length > 100 kb) from the NCBI nucleotide database (up to July, 2022)[6-29]. Meta data associated with the EBV genomes including the geographic origin, EBV type, carriers’ disease and collection date were also retrieved for all these sequences (Supplementary Table 2).

In order to combine multiple EBV genome sequences into a single dataset, we performed multiple sequences alignment merging public and private datasets (n=1477). Sequences were aligned against reference genome (NC_007605.1) using MAFFT[30] (v7.471) with the configuration ‘--keeplength’ to resolve the homology between the query and reference genome (171,823 bp). To improve the quality of aligned sequences, we masked repeat regions (annotated in NC_007605.1) by character ‘N’ and removed sequences with too many (>25%) non-canonical bases (not of “ATCG”) or deletions. In the end, 1334 EBV genome sequences were retained for further analysis (Supplementary Fig. 2). Single nucleotide variations (SNVs) for the aligned data were extracted using a custom python script. Subsequently, we used vcftools (v0.1.13)[31] to filter away variants with high missing values (more than 30%). We curated various subsets of data for different analyses by selecting subsets of strains from the global dataset (see Maintext).

**Principal component analysis and population structure analysis**

Smartpca from EIGENSOFT (v7.2.1)[32] was used to perform the principal component analysis (PCA). The PCA loadings of each single nucleotide polymorphism (SNP), reflecting the contribution to the principal components (PCs), were obtained from smartpca[32]. To visualize the variation in the PC loadings, we employed a sliding window approach (1000 bp windows with 500 bp step size) and summed the loadings for each window to depict the PC loadings across the genome. Population structure analysis was performed using Admixture (v1.3.0)[33] to estimate the population substructure and infer the genetic components of all individuals in our datasets. We used hierarchical clustering based on the Euclidean distance to plot the admixture results.

**Population history across humans and EBVs**

We first extracted regional populations with at least five EBV genomes and inferred the EBV population branching history based on the joint allele frequencies across populations using TreeMix (v1.13)[34]. Human variation data was collected from PGG.Han database[35], which included a large number of ethnic groups (Supplementary Table 3). SNPs in strong linkage disequilibrium were pruned using plink (v1.90, [www.cog-genomics.org/plink/1.9/](http://www.cog-genomics.org/plink/1.9/))[36] with parameters “--indep-pairwise 200 25 0.5”. Human population history was also inferred using TreeMix. In order to compare the branching history of humans and EBVs, we rooted both trees with the African populations and compared trees using cophylo function from R packages ape[37] and phytools[38].

**Recombination analysis and identification of the clonal strain**

Pairwise similarity was calculated using the ape package[37]. Pheatmap function in R was used for the clustering of sequences. Gubbins (v3.2.1)[39] was used for inferring recombination events in all the unique type 1 EBV genomes (n=1192). The genealogical tree was constructed by RAxML[40] integrated in Gubbins based on subsets of the sites after filtering away putative recombination regions. We defined the clonal strain as a group of highly similar and non-recombinant strains forming a monophyletic clade in the phylogenetic tree inferred from Gubbins.

We calculated the mean similarity between genomes from the clonal clade C1 to genomes outside of the clade based on a sliding window approach (1000 bp window size, 50 bp step size). The rank values of similarity for each window and the heatmap was plotted using a custom R script. For detecting recombination events, we obtained consensus genome sequences of the focal clades (e.g. S1, N1 and C1) using EMBOSS (v6.6.0.0)[41] and performed recombination analysis based on the consensus genomes using RDP5[42]. RDP5 integrates 7 different methods which includes RDP, GENECONV, Bootscan, Maxchi, Chimaera, Siscan and 3Seq as statistical methods for detecting recombination. Fst between samples (e.g. N1, S1 and C1) were calculated using vcftools (v0.1.13)[31].

**Association analysis**

The association between NPC and EBV subtypes was analyzed using a logistic regression model in a cohort of 337 NPC cases and 178 healthy controls from Guangdong or Hong Kong (Test 1 in Fig.2C). To further estimate the NPC risk associated with the clonal strain compared to the NC-HRS subtype, the logistic regression model was exclusively applied to the two specific EBV subtypes: NC-HRS and the clonal strain (Test 2 in Fig. 2C). The age-standardized incidence rate (ASR, per 100,000) data were collected from WHO-IARC and previous publications[43,44].

**Molecular dating**

Molecular dating was performed in BEAST (v2.7.5)[45]. Date-randomization tests (DRTs) were performed using R package TipDatingBeast. Since the BEAST analysis can only be conducted in small datasets. We performed random sampling of EBV genomes with known collection date, comprising of Africa (n=20), Europe (n=20), Asia (n=20), and additional genomes representing the clonal clade (n=10). In order to investigate the robustness of the estimates, we also conducted a large number of different subsampling (n=50) for different molecular dating. In particular, the analysis results of one subsample were presented in the maintext and results from additional replicates (n=50) were summarized in the Supplementary Figure 14.

For the molecular dating, we have chosen two clock models: strict clock and relaxed clock, and selected three demographic priors, including constant, exponential and Bayesian skyline. The Markov Chain Monte Carlo (MCMC) steps were set to be between 30~120 million to ensure convergence (e.g. effective sample size (ESS) >200 for the parameters and were manually checked with tracer[46]). The EBV genomes of a 5700 years-old sample were obtained from a piece of chewed birch pitch[47]. We downloaded the sequenced reads, mapped the reads to EBV reference genome (NC_007605) using BWA-MEM and obtained the subset of the genome with at least a depth of 2 (length=3775 bp).

**Selection analysis**

The folded site frequency spectrum (SFS) was calculated based on the frequencies of the biallelic single nucleotide polymorphism in different geographic populations (i.e. Hong Kong and Guangdong). The expected SFS is computed under the neutral expectation. The Fu and Li’s test was performed using PopGenome[48] and pegas[49] packages in R. The neutral distribution of Fu and Li’s F* and D* were calculated from the neutral simulations using ms[50].

In order to estimate the selective coefficient of the recombinant virus, we employed a haploid selection model from Population Genetics [51]. In particular, we assume a human population with effective population size 10,000. When the recombinant virus first arises, it only infected one individual with an initial frequency of 1/10,000. We assume the recombinant virus has a fitness value 1+s, where s is the selective coefficient. Under the haploid selection model, the recombinant allele will increase in frequency for a given time span. Using population genetic theory, we can get an estimate of the selection coefficient using the haploid selection model[51]. To be more precise, the allele frequency of the selected allele through time can be expressed as:

$$f_{t}=\frac{f_{0}}{f_{0}+\left( \frac{1}{1+s} \right)^{t}(1-f_{0})}$$

Where f_0_ is the initial frequency, f_t_ is the frequency of the selected allele at generation t.

**Contribution of different EBV subtypes to the NPC risk**

The contribution of EBV strains to the NPC risk was evaluated by population attributable fraction (PAF) and NPC prevalence in response to the estimated frequency trajectory of the clonal strain over time. Firstly, using NPC cases and controls from southern Chinese, we assessed the OR of NPC for the clonal strain (11.46, 95% CI=7.13-18.43) and the NC-HRS (6.19, 95% CI=3.38-11.34), respectively, compared to the other EBV strains as a reference group by the logistic regression model. Based on the different frequencies of the clonal strain over time, the PAF of NPC risk explained by the effect of the clonal strain, was modelled as follows[52]:

$${PAF}_{Clonal}= \frac{F_{Clonal}\times({RR}_{Clonal}-1)}{1+F_{NC-HRS}\times({RR}_{NC-HRS}-1)+F_{Clonal}\times({RR}_{Clonal}-1)}$$

$${PAF}_{NC-HRS}= \frac{F_{NC-HRS}\times({RR}_{NC-HRS}-1)}{1+F_{NC-HRS}\times({RR}_{NC-HRS}-1)+F_{Clonal}\times({RR}_{Clonal}-1)}$$

, where $F_{NC-HRS}$ represents the frequency of the NC-HRS, and $F_{clonal}$ represents the frequency of the clonal strain in healthy population, and $RR$ is the relative risk of NPC associated with exposure to the NC-HRS or the clonal strain of EBV. As NPC is a rare disease with a prevalence of 0.001625[53], the relative risk (RR) can be approximated by the OR. As the frequency of clonal strain ($F_{clonal}$) increased in the population, the NC-HRS and the reference group were replaced proportionally to their current frequencies. The 95% CI for the PAF is estimated using the delta method based on the CIs of OR associated with the clonal strain and the NC-HRS.

For the estimation of NPC prevalence in response to the population trajectory of the clonal strain over time, firstly, we constructed a logistic regression model to represent the association between NPC and two EBV subtypes:

$$Logit P= \beta_{0}+\beta_{1}\times X_{NC-HRS}+\beta_{2}\times X_{Clonal}$$

, where $P$ is the individual probability of developing NPC, and $X$ is the categorical variable indicating infection with the NC-HRS or the clonal strain. We estimated the parameter $\beta_{1}$ and $\beta_{2}$ in this logistic regression model based on NPC cases and controls from southern Chinese in our dataset. Thus, for individuals infected with EBV from the reference group, the probability of NPC is $P_{ref}= \frac{1}{1+ e^{-\beta_{0}}}$, for individuals infected with NC-HRS, the probability of NPC is $P_{NC-HRS}= \frac{1}{1+ e^{（- \beta_{0} -\beta_{1}）}}$, and for individuals infected with clonal strain, the probability of NPC is $P_{Clonal}= \frac{1}{1+ e^{（- \beta_{0} -\beta_{2}）}}$. Then, the prevalence of NPC can be calculated based on the frequency of clonal strain as follows:

$$Prevalence of NPC= {F_{ref}\times P_{ref}+F}_{NC-HRS}\times P_{NC-HRS}+ F_{Clonal}\times P_{Clonal}$$

, where $F_{ref}$ is the frequency of the reference group, $F_{NC-HRS}$ is the frequency of the NC-HRS and $F_{Clonal}$ is the frequency of the clonal strain in the population. Given the NPC prevalence of 0.001625 [53] and the frequencies of clonal strain, NC-HRS, and the reference group (0.309, 0.135, and 0.556, respectively, observed in healthy individuals from southern Chinese in our dataset), we estimated the parameter $\beta_{0}$ in the logistic regression model. As the frequency of clonal strain ($F_{clonal}$) increased in the population, the NC-HRS and the reference group were replaced proportionally to their current frequencies. The prevalence of NPC associated with the clonal strain was calculated as $F_{Clonal}\times P_{Clonal}$, while the prevalence of NPC associated with the non-clonal strain was calculated as ${F_{ref}\times P_{ref}+F}_{NC-HRS}\times P_{NC-HRS}$. The 95% CI was estimated using the delta method based on the CIs of $\beta_{1}$ and $\beta_{2}$.

**Supplementary Notes 1. Sampling bias in the public dataset and its potential effect on the evolutionary analysis**

As EBV can cause a wide range of diseases with strong geographic preference, the available sequences in the public databases also reflect this biased distribution. For example, NPC is exceptionally frequent in southern China, and endemic Burkitt lymphoma is very frequent in sub-Saharan Africa, where malaria is hyperendemic. The question is, how different sample size and disease types can affect the evolutionary analysis. In the maintext, we have subsampled equal number of sequences from different geographic locations and performed structure analysis on the subsample, we observed a similar pattern of population substructure. In order to further address this question, we explored a few directions:

1) We compared the EBV genomes derived from saliva (normal) and disease tissue (i.e. NPC) from the same individual (paired samples), we found that the strains are highly consistent compared to strains from different individuals[28]. Thus, the EBV genomes from healthy (e.g. saliva) or disease tissues would likely to be derived from the same EBV viral population in the humans of the specific geographic location.

2) We take advantage of the fact that there are a small number of EBV genomes sampled from healthy individuals (mainly from Europe and Asia). We picked healthy samples from Europe and Asia representing the EBVs from these two regions. When we perform the structure analysis based on the healthy individuals only, we found that the results of structure analysis are similar with that when we use all samples (Supplementary Figure 15 and Supplementary Figure 6 and Figure 7).

3) In order to explore the relationship between healthy/disease samples from the same geographic regions, we conducted additional analysis to compare pairwise genetic distances of EBV genomes (disease or healthy) between and within different populations. Our intuition is that, EBV genomes (disease or healthy) from the same geographic regions should be much more similar than EBV genomes from different geographic regions (i.e. population substructure, Supplementary Figure 16). The results suggest that genetic distances between healthy and disease samples from the same regions are a lot smaller than genetic distances calculated between regions (i.e supporting the population substructure).

Taken together, we think many evolutionary analyses are not strongly affected by the sampling biases.


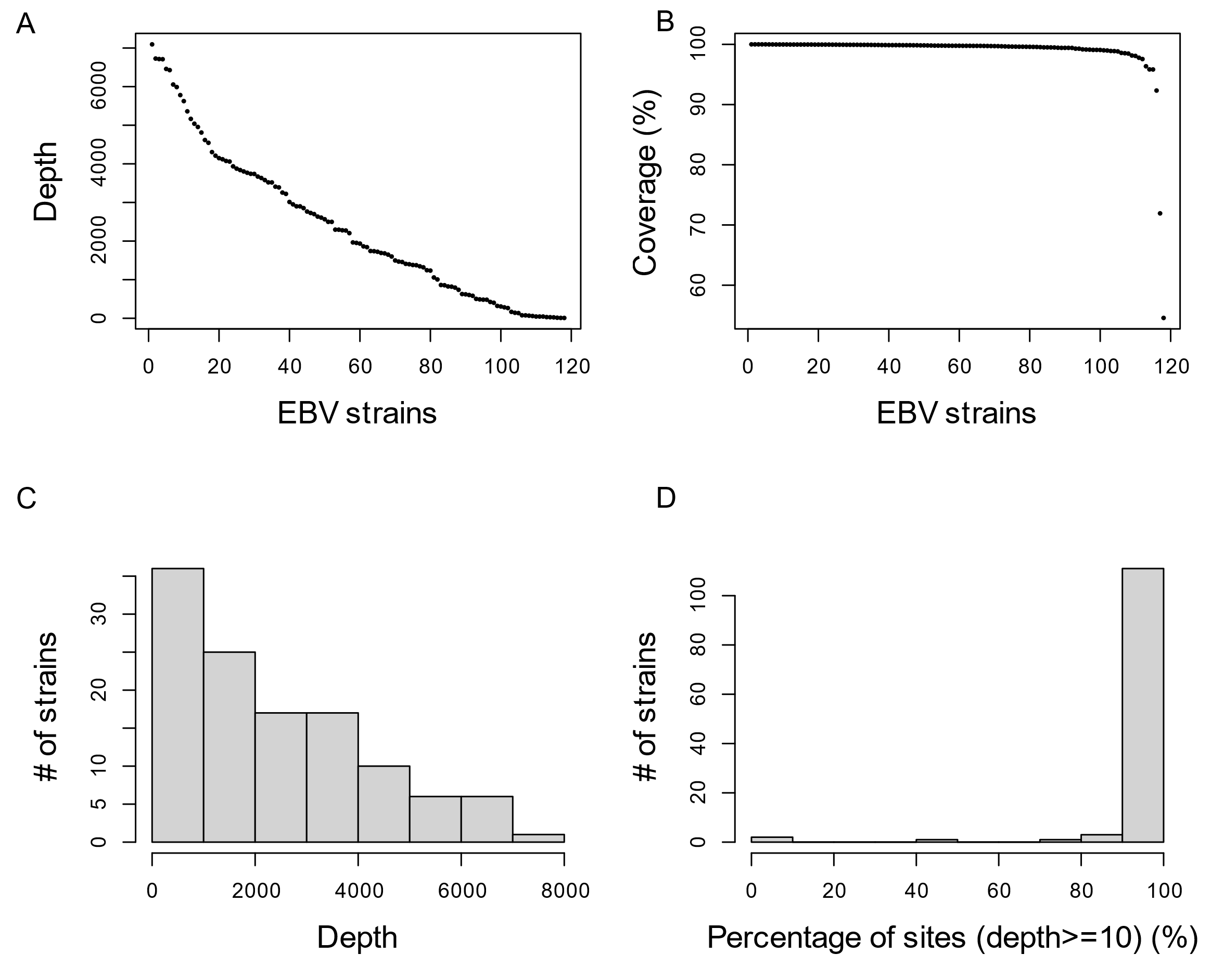


Fig. S1. Summary of the 118 sequenced EBV genomes. A) Sequencing depth across the 118 genomes and B) Percentage of the genome covered across 118 EBV genomes. C) Distribution of sequencing depth across the 118 genomes. D) Percentage of sites whose sequencing depth are greater than 10 across all the 118 genomes.


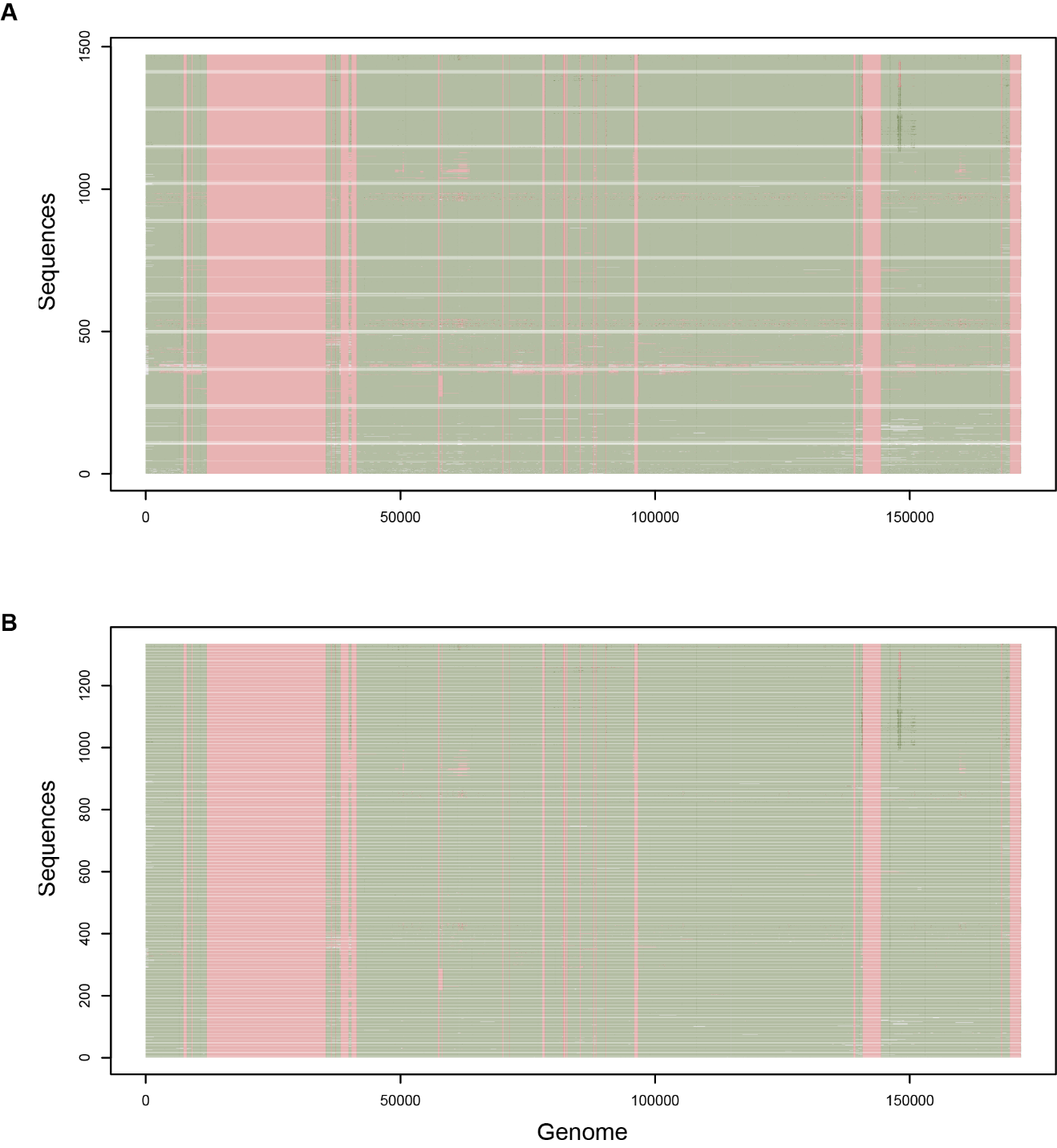


Fig. S2. Overall landscape of the multiple sequence alignments. A) Raw alignment based on 1472 aligned sequences. B) alignment of the remaining 1334 genomes after quality control. The repeat regions of genomes were masked as letter ‘N’. Green dots stand for normal bases i.e., ATCG; while red dots representing ambiguous bases ‘N’ (often in repeat regions) and grey ones are deletions.


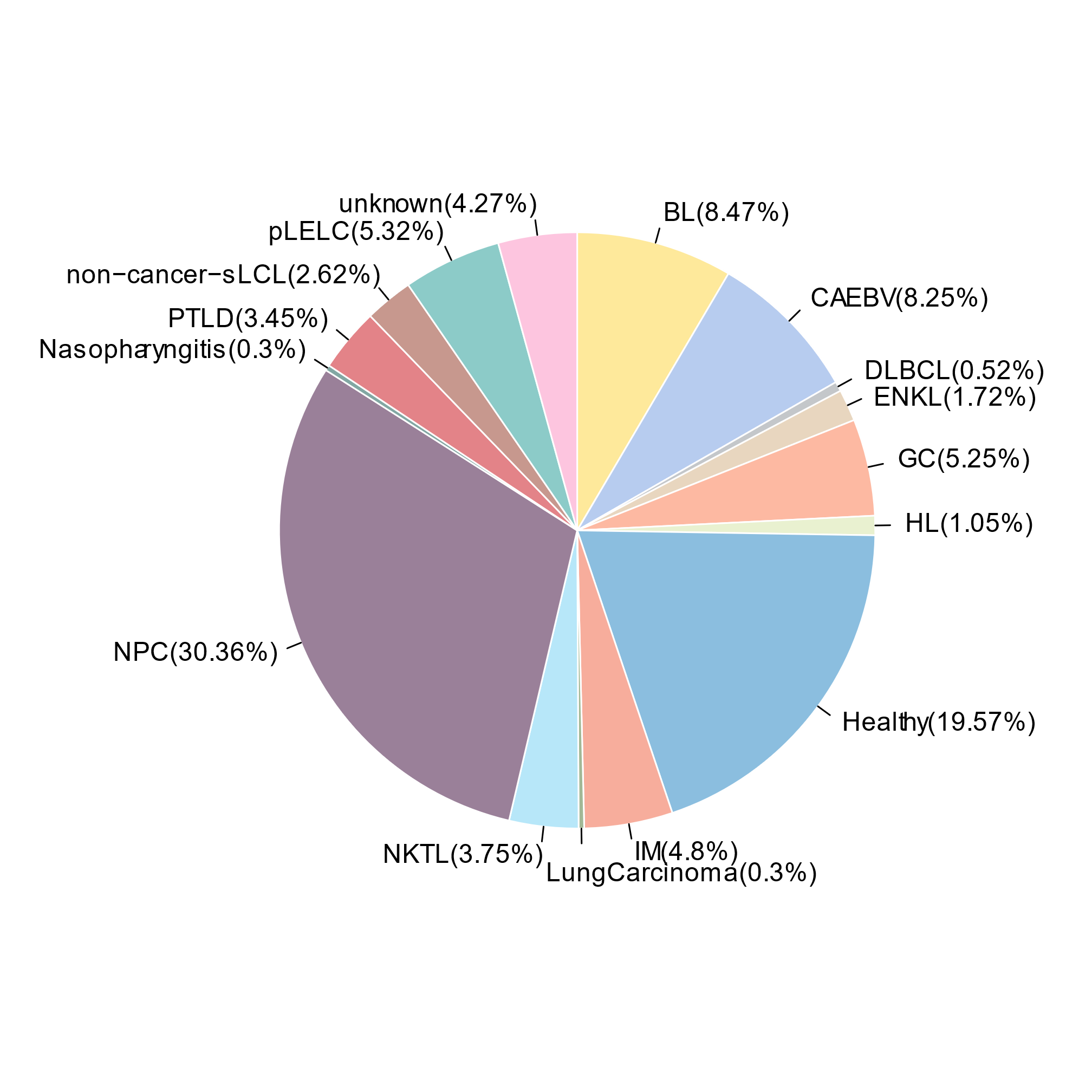


Fig. S3. Health status of the EBV carriers. Pie chart shows the distribution of health status of the carriers, most of which are NPC and healthy individuals. NPC (nasopharyngeal carcinoma), BL (Burkitt lymphoma), HL (Hodgkin lymphoma), IM (infectious mononucleosis), PTLD (posttransplant lymphoproliferative disease), GC (gastric carcinoma), CAEBV (chronic active EBV), DLBCL (diffuse large B cell lymphoma), ENKL (extranodal natural killer / T-cell lymphoma, nasal type), NKTL (NK/T cell lymphoma), pLELC (Primary pulmonary lymphoepithelioma-like carcinoma).


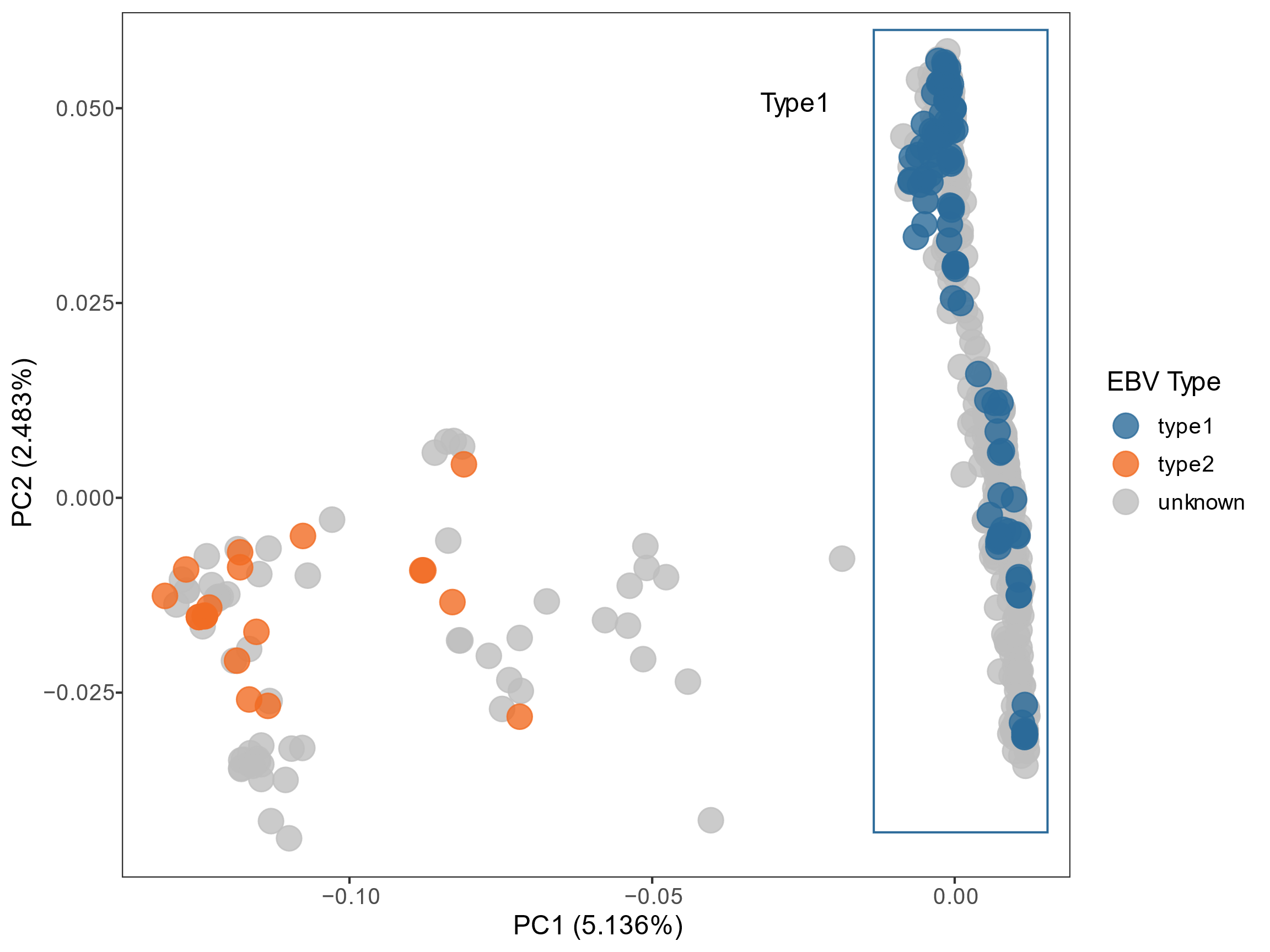


Fig. S4. Principal component analysis of all EBVs. First two principal components (PCs) are plotted on the two axes. Stains with known subtypes of EBVs labeled as blue (type 1) and orange (type 2) symbols, even though most of the strains have no subtype information. We selected all sequences in the blue rectangle as type I sequences.


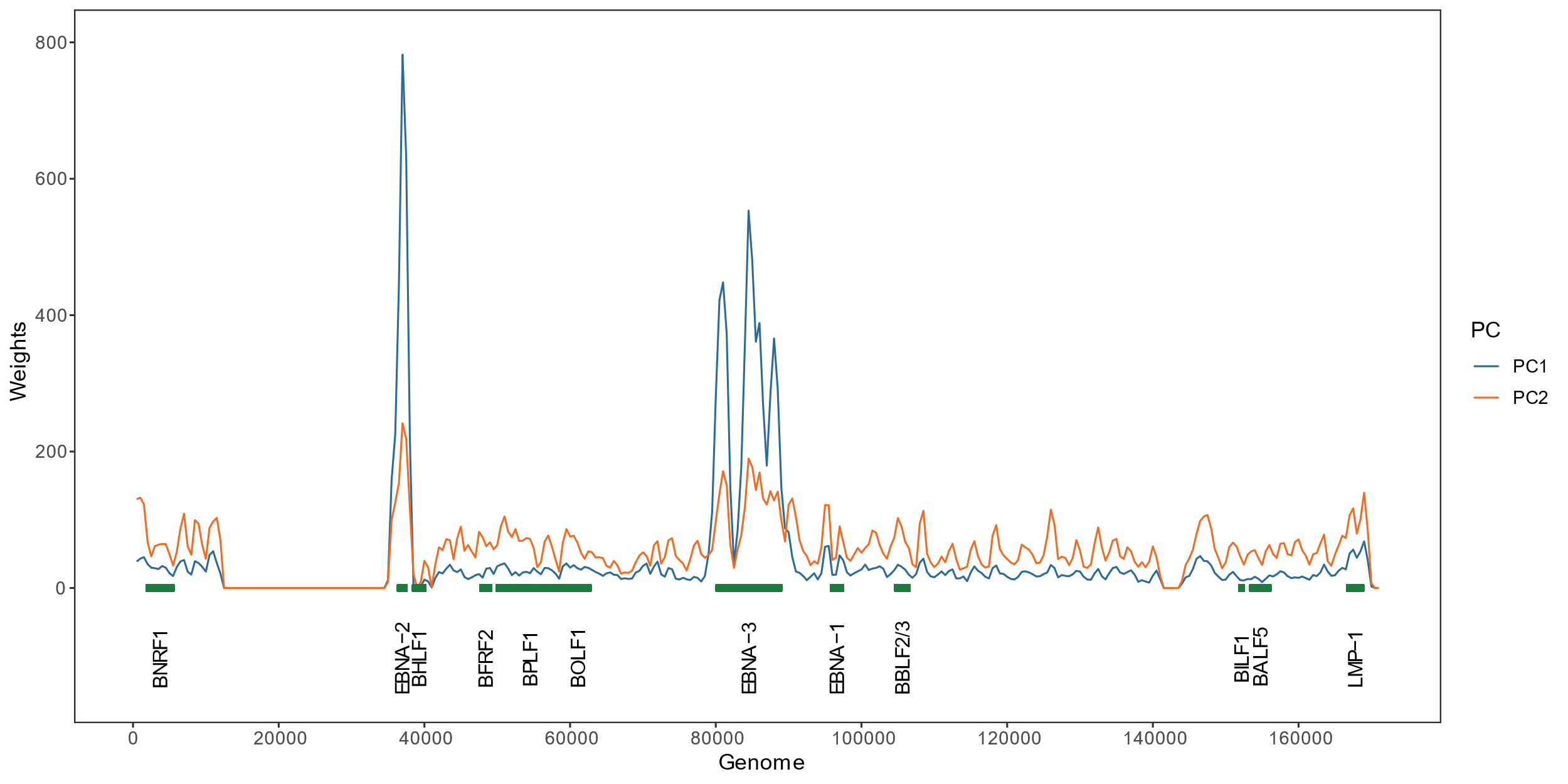


Fig. S5. PC loadings across the genome. The PC loadings (y-axis) contributed by different genomic regions were plotted across the EBV genome. CDS of specific genes are labeled underneath the PC loadings.


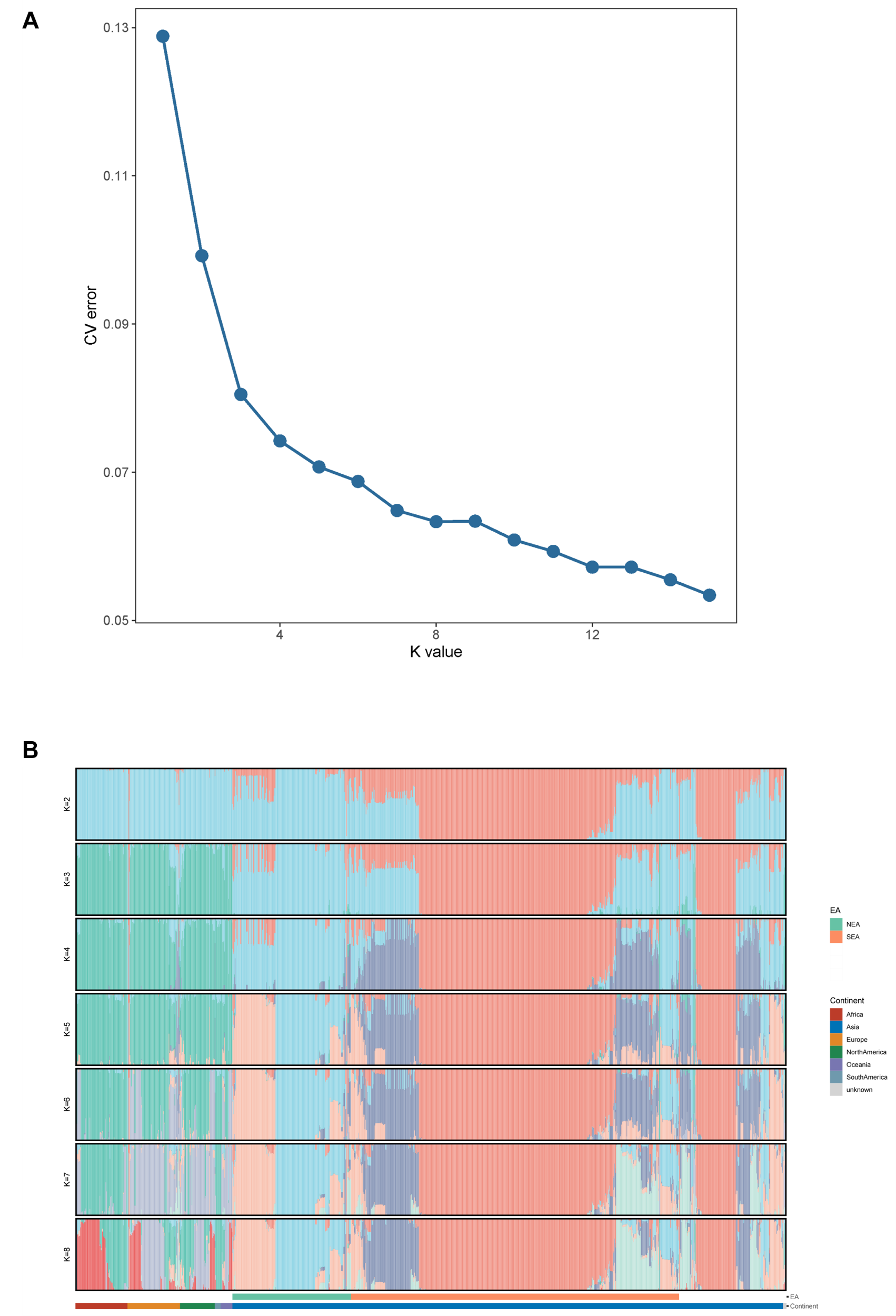


Fig. S6. Population structure analysis. (A) CV (Cross-Validation) errors across the admixture analysis when analyzing 1205 type 1 EBV strains with varying numbers of population groups (k=2-15) (B) Populations structure of type 1 strains. Different ancestries (K) are labelled by different colors. The geographic locations of the EBV carriers were labelled underneath the structure plot.


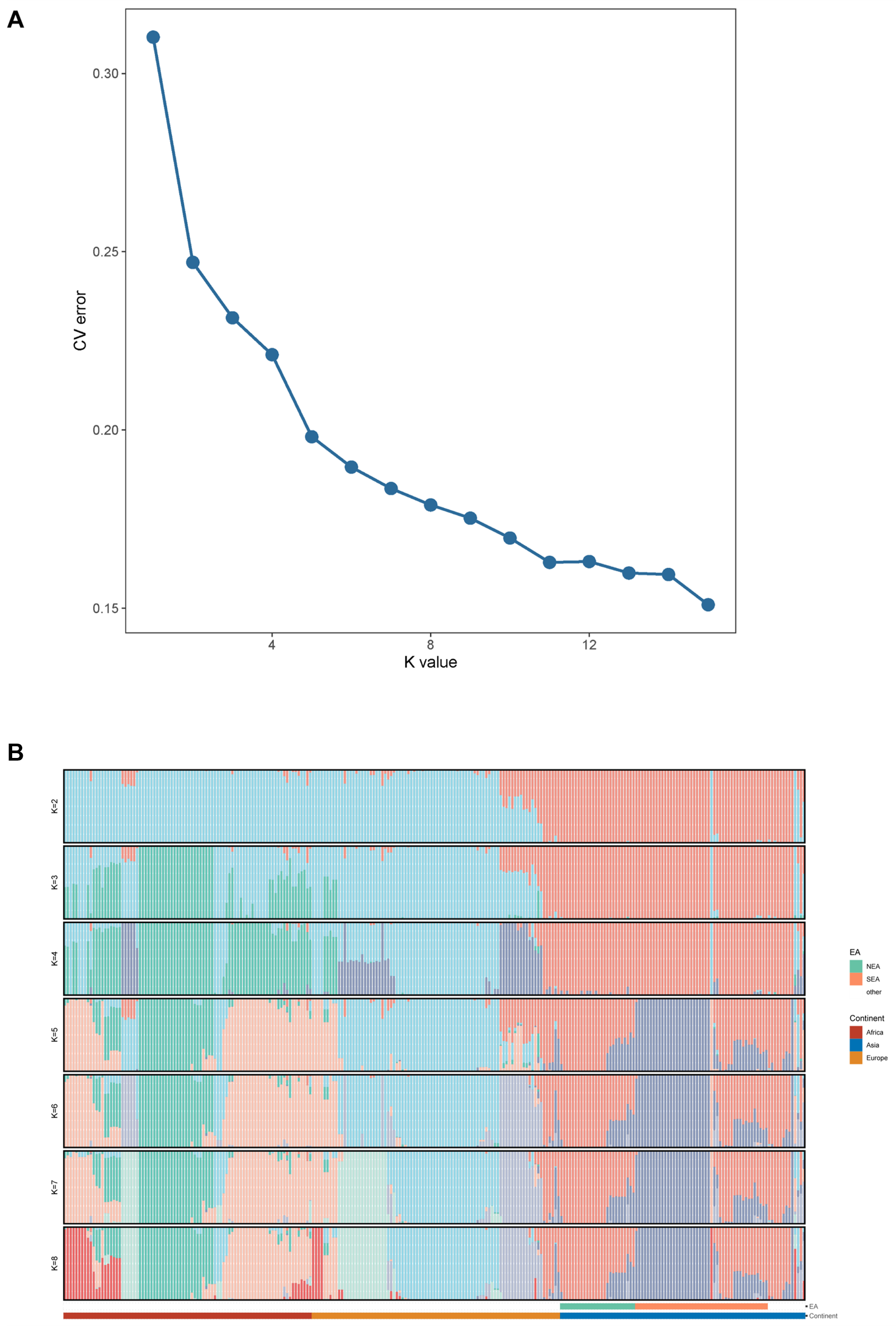


Fig. S7. Population structure analysis. (A) CV (Cross-validation) errors in the admixture analysis of the balanced dataset where similar numbers of EBV strains were subsampled from different geographic locations to generate the balanced dataset. CV values were plotted across different K (number of subgroups). (B) Population structure analysis of the balanced dataset. Different ancestries (K values) and geographic locations were annotated similar to Figure S6.


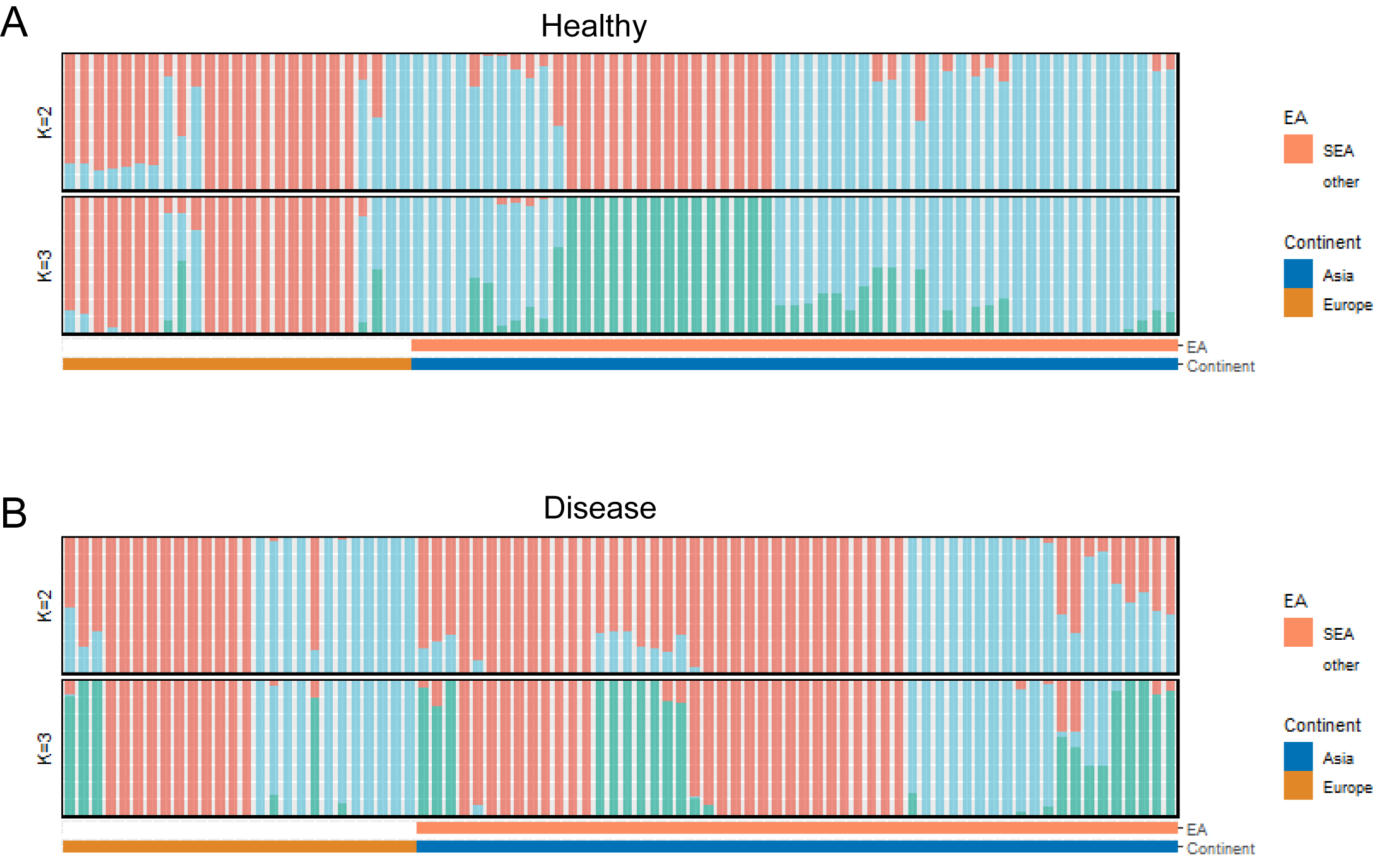


Fig. S8. The structure analysis using healthy and disease samples for Europe and Asia. A) Structure analysis using only healthy samples, B) Structure analysis using disease samples only.


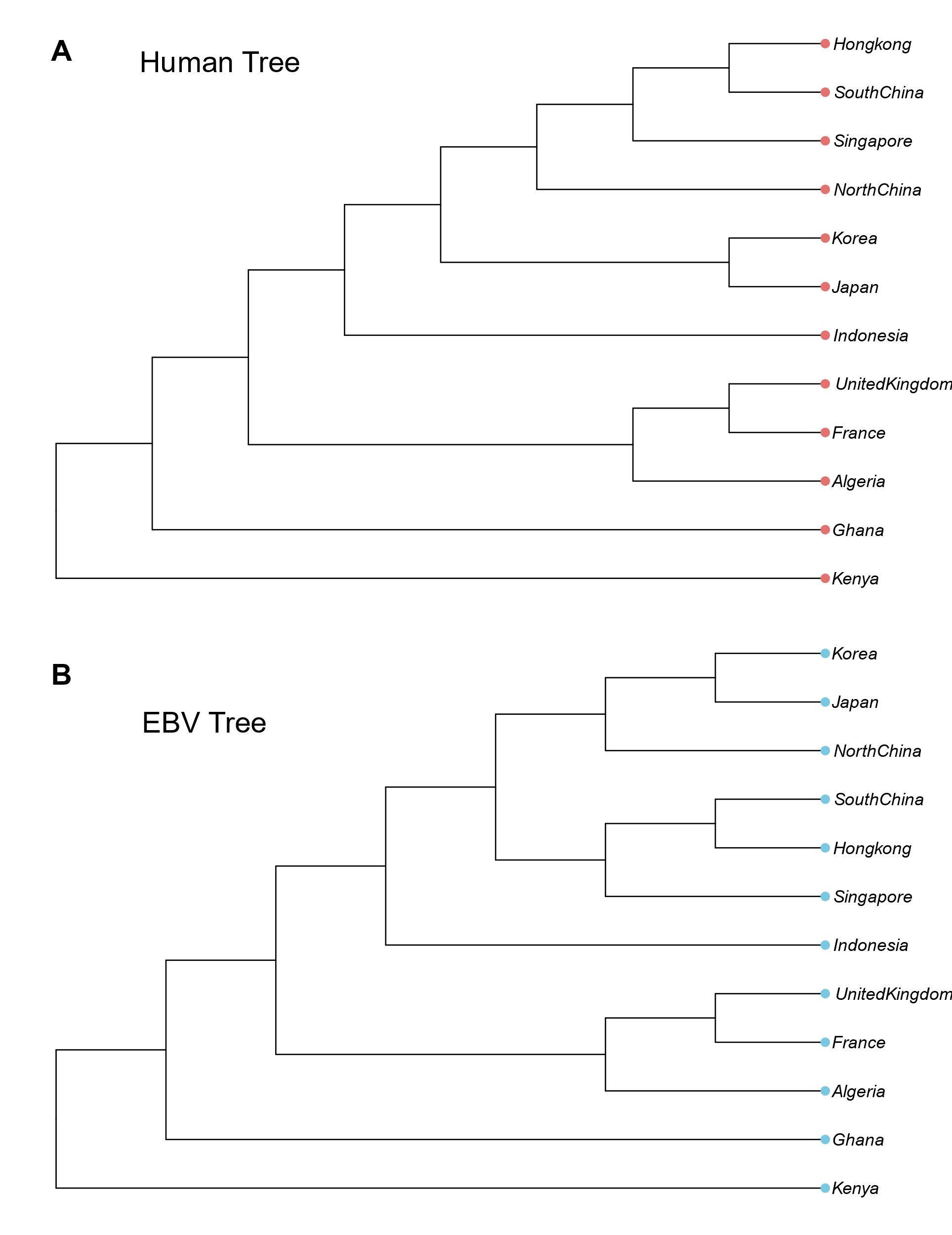


Fig.S9. Phylogenetic tree of Human and EBV geographic populations. (A) Human population tree (topological relationship). (B) EBV population tree (topological relationship).

**
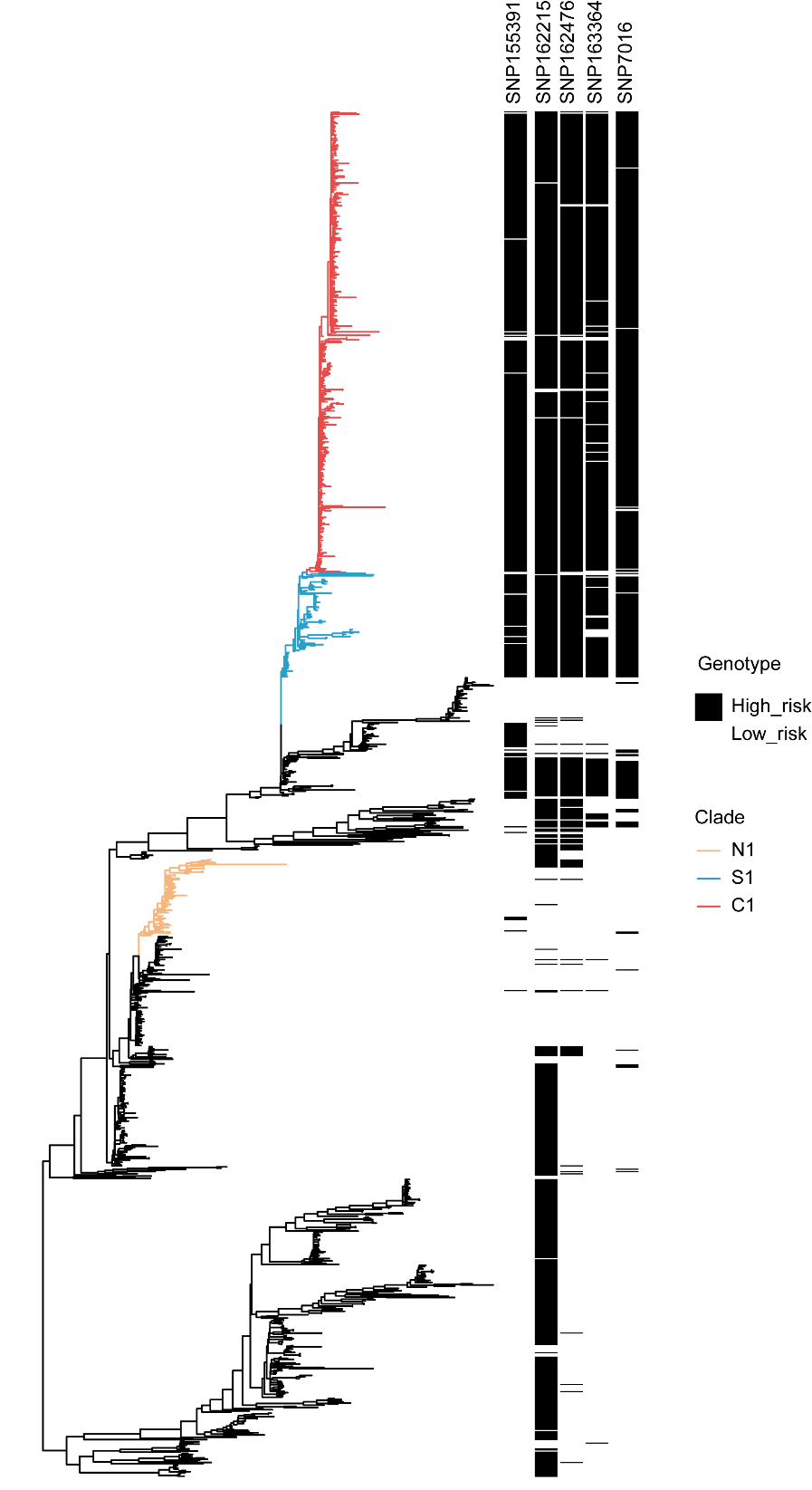
**

Fig. S10. High-risk variants associated with NPC risk identified in previous studies. SNP155391 was identified in 2015[54], SNP162215, SNP162476 and SNP1663364 were identified in 2019[28], SNP7016 was identified in 2018[25].


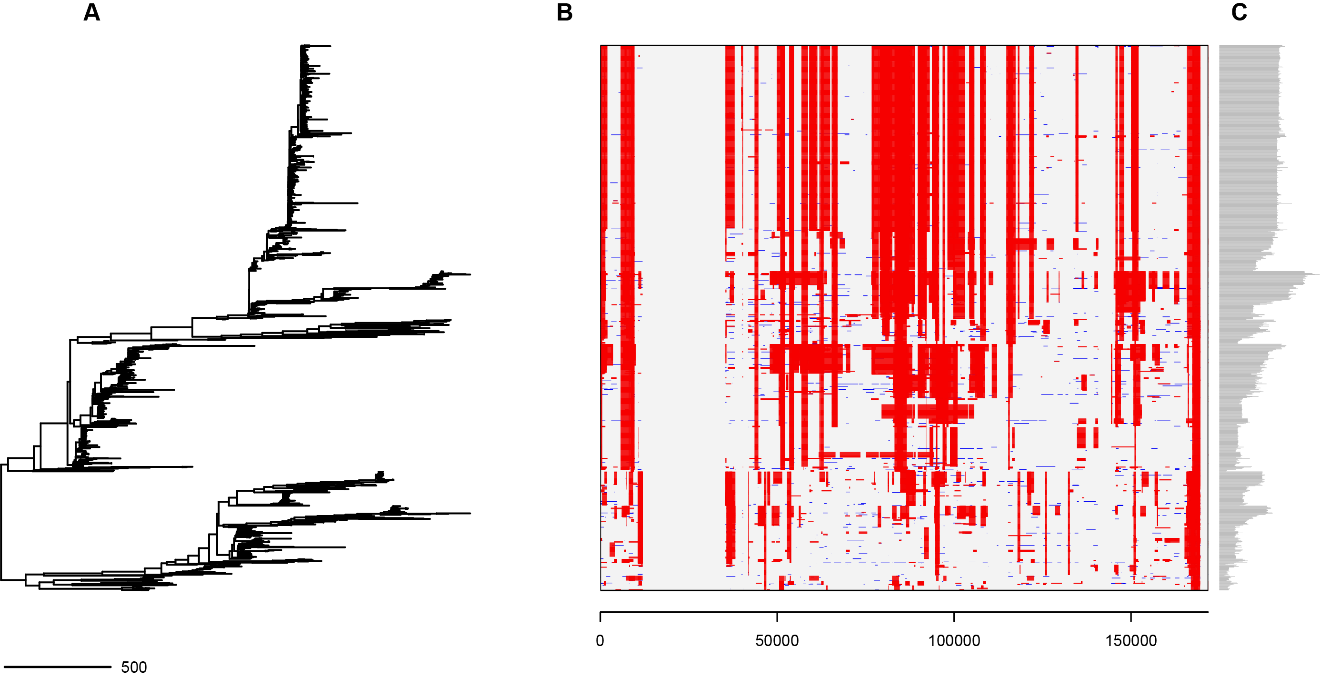


Fig. S11. Recombinant landscape inferred by Gubbins. (A) Maximum likelihood phylogenetic tree reconstructed after masking putative recombinant regions. ML tree was constructed by RAxML embedded in Gubbins. (B) Inferred recombinant events. Columns represent the bases along the genome. Rows indicates the strains corresponding to the tips in phylogeny (left). Red blocks indicate putative recombinant events occurring on internal nodes, while blue ones indicate recombination events occurring on terminal branches. (C) Bar plot of the number of recombinant sites occurring in each sequence.


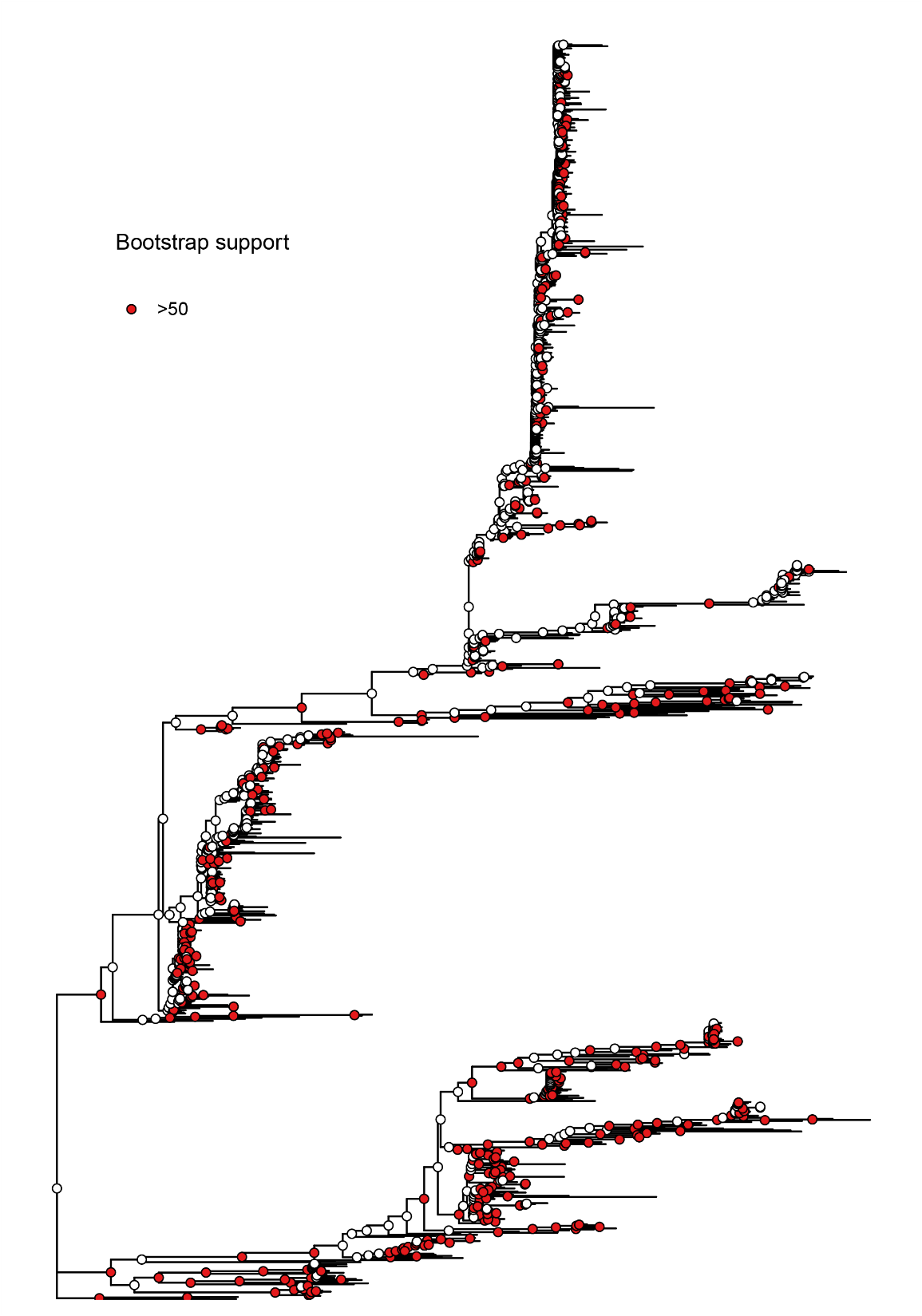


Fig. S12. Bootstrap of the reconstructed phylogenetic tree from Gubbins. Red circles indicate nodes supported by a bootstrap value >50%.


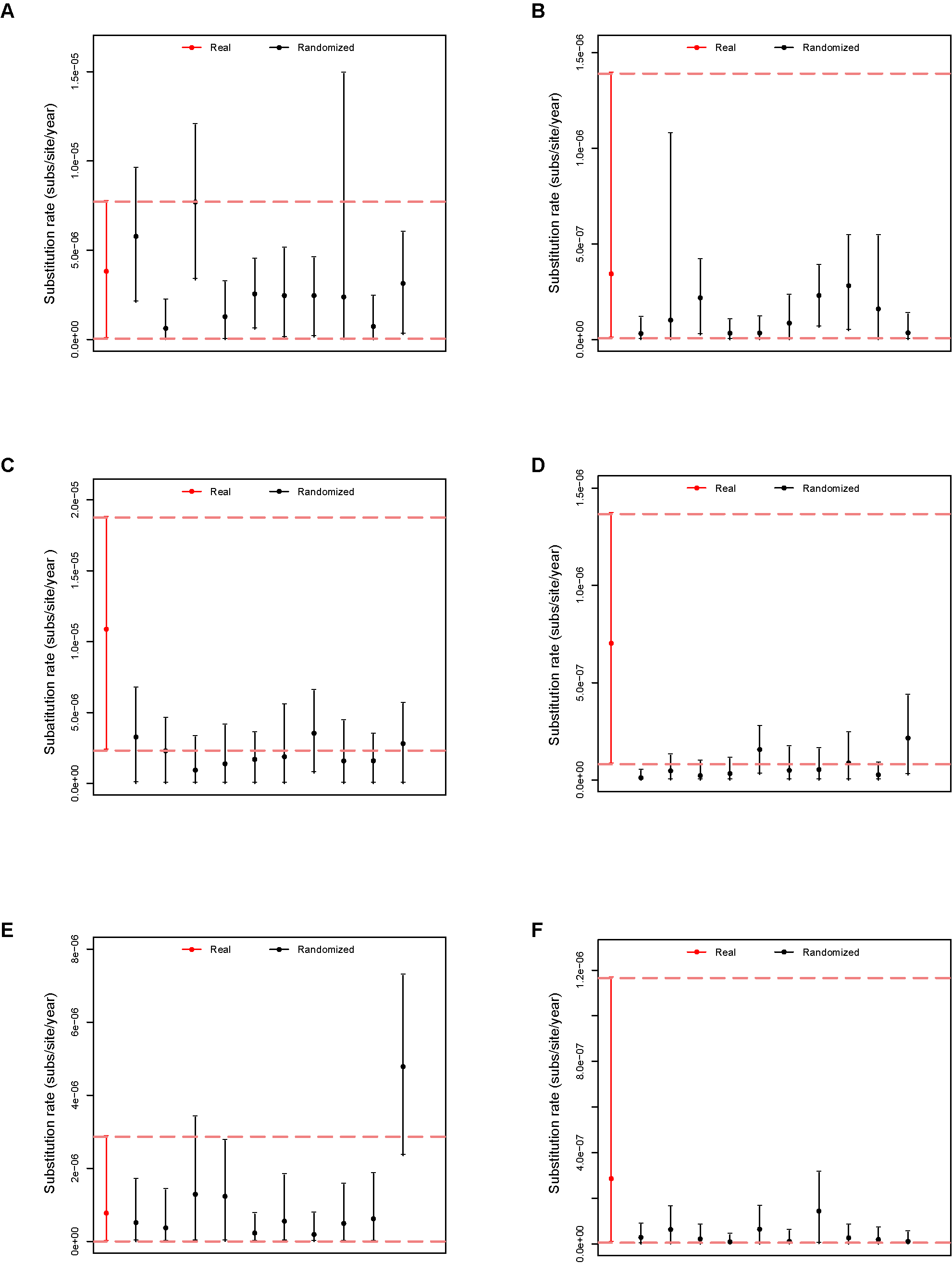


Fig. S13. Date randomization test. A) randomization test for the subsample presented in Figure 4A(maintext), B) randomization test when combining the subsample and the ancient sample. Red lines are the estimated time intervals from real data, while black lines are estimates from randomly-sampled data where dates were randomly shuffled among the sequences.


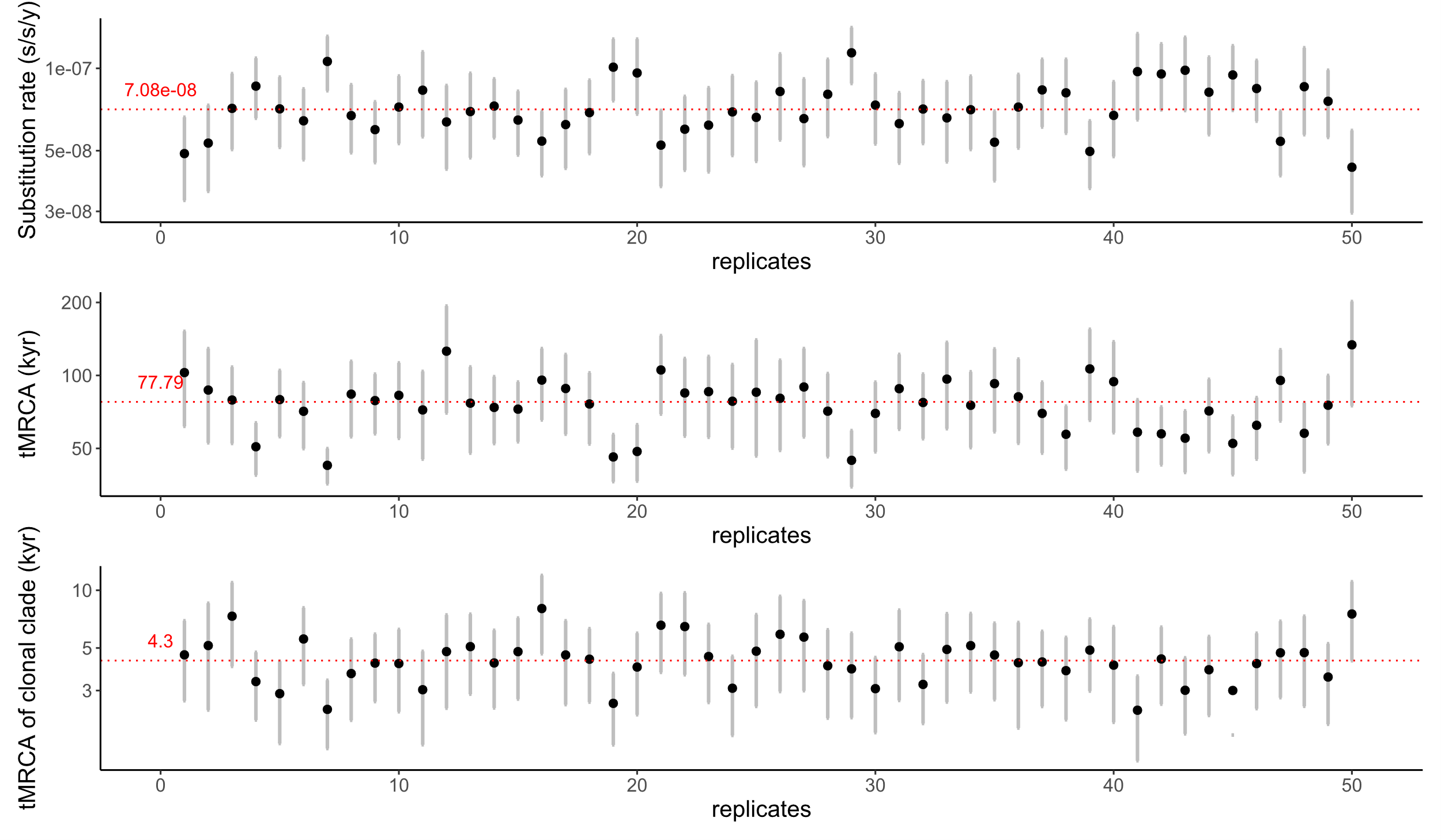


Fig. S14. BEAST output for 50 subsamples. 50 random subsamples (with 20,20,20 and 10 sequences from Africa, Europe, Asia (nonclonal) and clonal clade) of EBV genomes were selected to perform molecular dating. The boxplots for substitution rate, tMRCA of all type 1 EBVs and tMRCA of the clonal clade were shown and median values across 50 replicates were labeled as the red line.


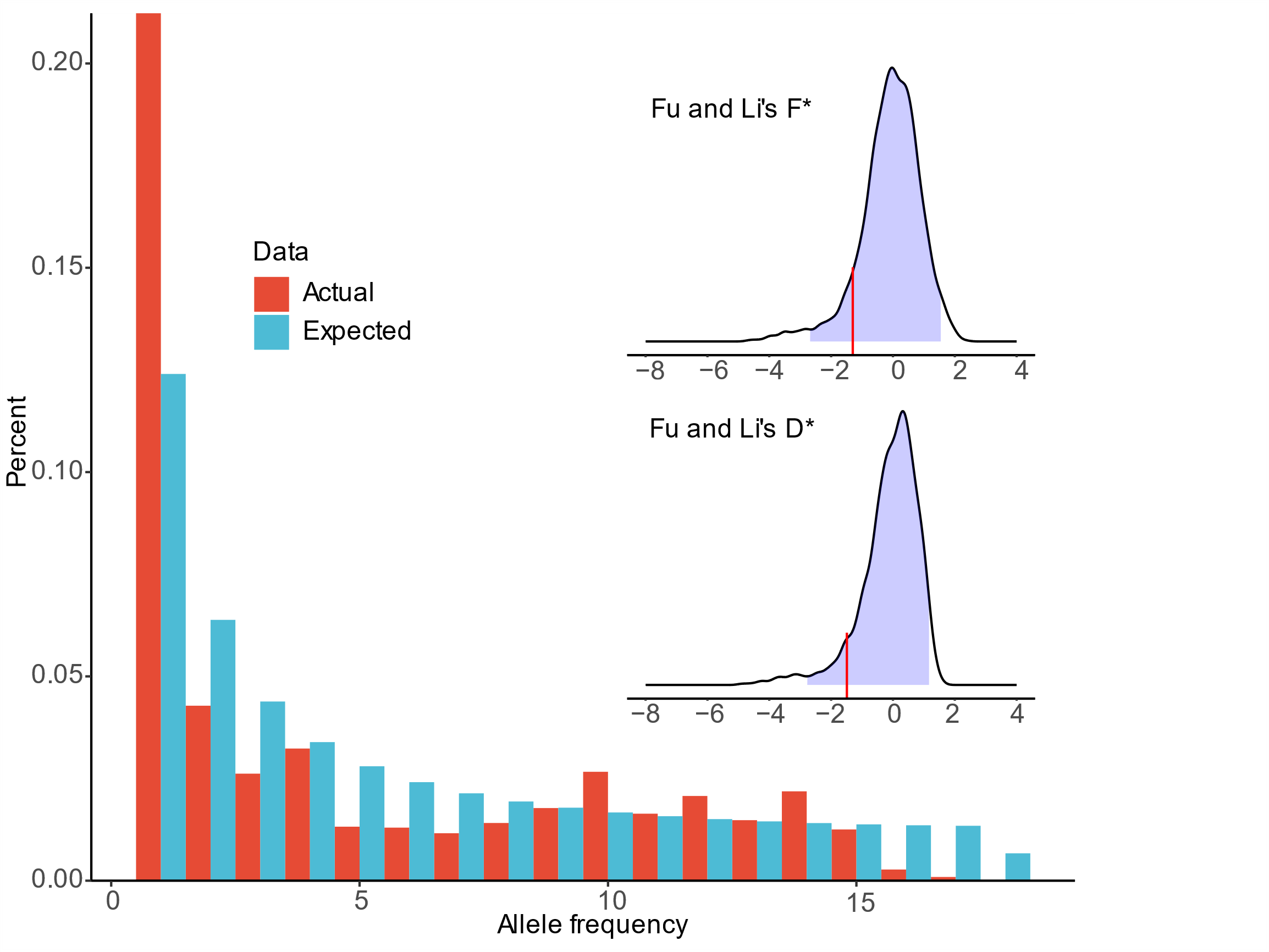


Fig. S15. Selection analysis of Guangdong population. The SFS and Fu and Li’s test on the Guangdong population (n=36). P-values for the D* and F* are 0.07 and 0.09 respectively.


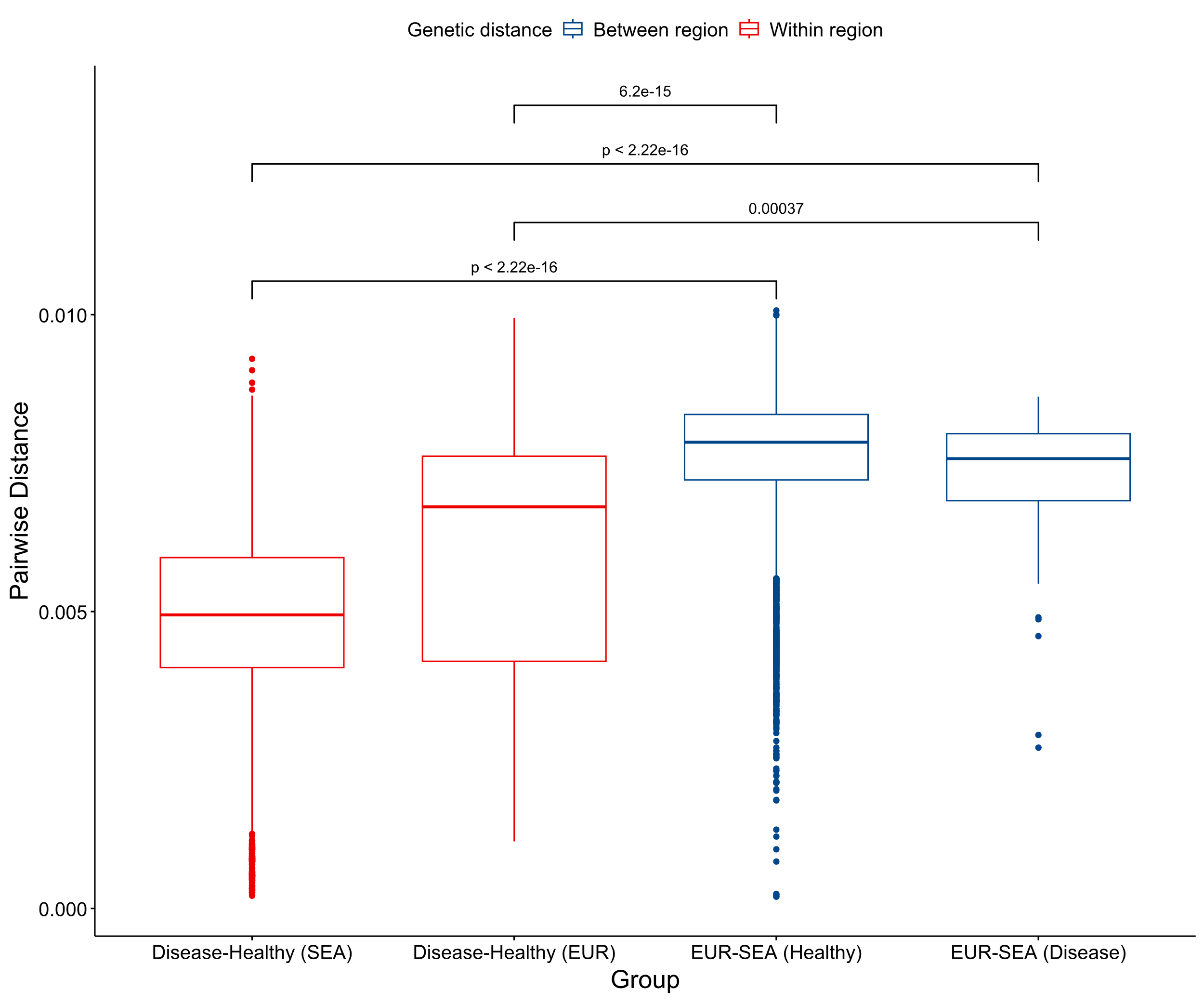


Fig. S16. The genetic difference between healthy and disease samples from different geographic areas. Boxplot showing the pairwise comparison between disease and healthy samples from the same region (red boxplot, SEA: South East Asia, EUR: Europe) as well as between different geographic regions (blue boxplot).


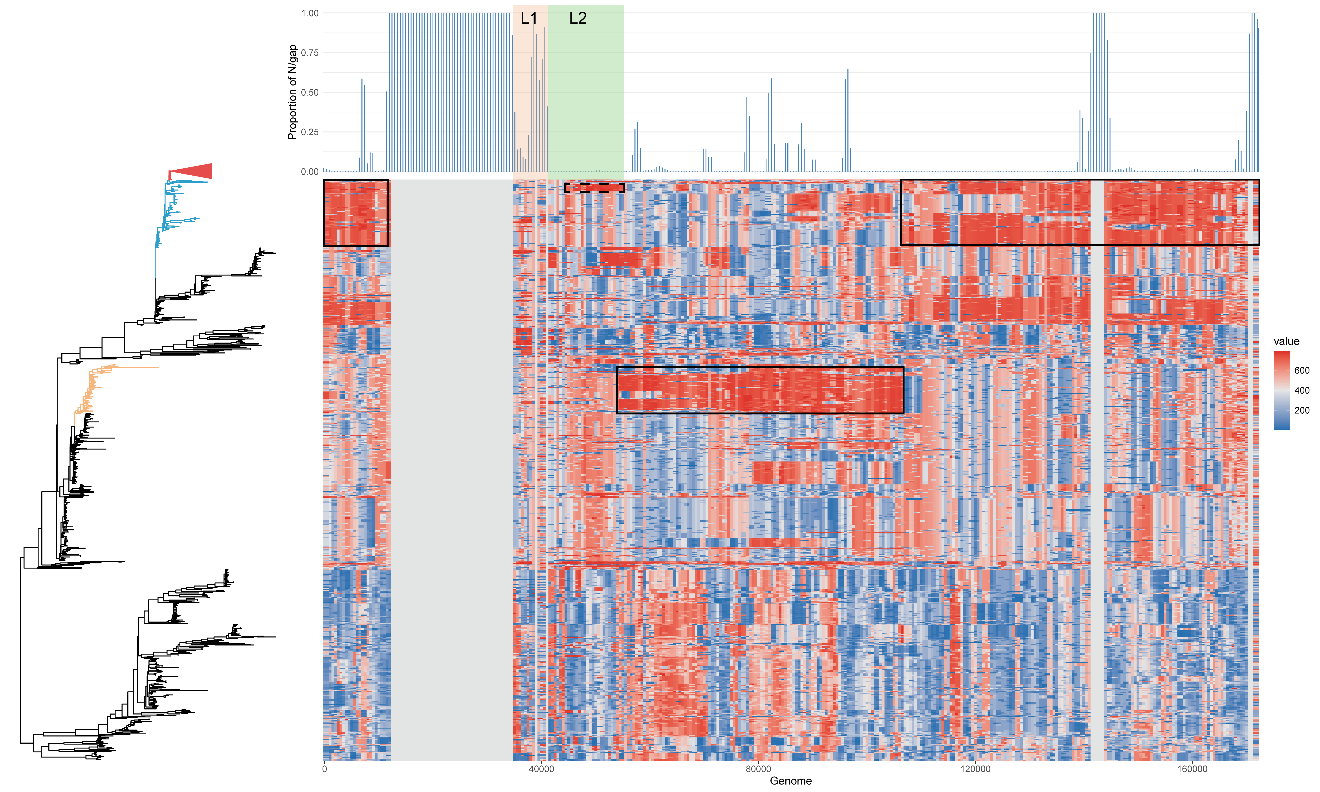


**Fig. S17. Missing parental origin around repetitive regions.** In our analysis, we didn’t find a clear parental source for the region to the right side of the large repetitive region. When we calculated the content of N (missing base) and gap along the genome, we found that there is a large region (denoted as L1) with high levels of missing bases (N or gap) which could lead to poor sequence comparison. In the second region (denoted as L2), we identified a potential parental source (in dashed black square) within the S1 clade.

**References**

1. Martin M. Cutadapt removes adapter sequences from high-throughput sequencing reads. *EMBnetJournal*. 2011;17(1):10-12.

2. Li H. Aligning sequence reads, clone sequences and assembly contigs with BWA-MEM. *arXiv:13033997v2*. 2013;

3. Li H, Handsaker B, Wysoker A, et al. The Sequence Alignment/Map format and SAMtools. *Bioinformatics*. Aug 15 2009;25(16):2078-9. doi:10.1093/bioinformatics/btp352

4. Poplin R R-RV, DePristo MA, Fennell TJ, Carneiro MO, Van der Auwera GA, Kling DE, Gauthier LD, Levy-Moonshine A, Roazen D, Shakir K, Thibault J, Chandran S, Whelan C, Lek M, Gabriel S, Daly MJ, Neale B, MacArthur DG, Banks E. . Scaling accurate genetic variant discovery to tens of thousands of samples. *bioRxiv*. 2017;201178

5. Van der Auwera GA, Carneiro MO, Hartl C, et al. From FastQ data to high confidence variant calls: the Genome Analysis Toolkit best practices pipeline. *Curr Protoc Bioinformatics*. 2013;43(1110):11 10 1-11 10 33. doi:10.1002/0471250953.bi1110s43

6. de Jesus O, Smith PR, Spender LC, et al. Updated Epstein-Barr virus (EBV) DNA sequence and analysis of a promoter for the BART (CST, BARF0) RNAs of EBV. *J Gen Virol*. Jun 2003;84(Pt 6):1443-1450. doi:10.1099/vir.0.19054-0

7. Zeng MS, Li DJ, Liu QL, et al. Genomic sequence analysis of Epstein-Barr virus strain GD1 from a nasopharyngeal carcinoma patient. *J Virol*. Dec 2005;79(24):15323-30. doi:10.1128/JVI.79.24.15323-15330.2005

8. Dolan A, Addison C, Gatherer D, Davison AJ, McGeoch DJ. The genome of Epstein-Barr virus type 2 strain AG876. *Virology*. Jun 20 2006;350(1):164-70. doi:10.1016/j.virol.2006.01.015

9. Liu P, Fang X, Feng Z, et al. Direct sequencing and characterization of a clinical isolate of Epstein-Barr virus from nasopharyngeal carcinoma tissue by using next-generation sequencing technology. *J Virol*. Nov 2011;85(21):11291-9. doi:10.1128/JVI.00823-11

10. Kwok H, Tong AH, Lin CH, et al. Genomic sequencing and comparative analysis of Epstein-Barr virus genome isolated from primary nasopharyngeal carcinoma biopsy. *PLoS One*. 2012;7(5):e36939. doi:10.1371/journal.pone.0036939

11. Lei H LT, Hung GC, Li B, Tsai S, Lo SC. Identification and characterization of EBV genomes in spontaneously immortalized human peripheral blood B lymphocytes by NGS technology. *BMC Genomics*. 2013;14:804.

12. Lin Z, Wang X, Strong MJ, et al. Whole-genome sequencing of the Akata and Mutu Epstein-Barr virus strains. *J Virol*. Jan 2013;87(2):1172-82. doi:10.1128/JVI.02517-12

13. Tsai MH, Raykova A, Klinke O, et al. Spontaneous lytic replication and epitheliotropism define an Epstein-Barr virus strain found in carcinomas. *Cell Rep*. Oct 31 2013;5(2):458-70. doi:10.1016/j.celrep.2013.09.012

14. Tso KK-Y, Yip KY-L, Mak CK-Y, et al. Complete genomic sequence of Epstein-Barr virus in nasopharyngeal carcinoma cell line C666-1. *Infect Agent Cancer*. 2013;8(1):29.

15. Kwok H, Wu CW, Palser AL, et al. Genomic diversity of Epstein-Barr virus genomes isolated from primary nasopharyngeal carcinoma biopsy samples. *J Virol*. Sep 2014;88(18):10662-72. doi:10.1128/JVI.01665-14

16. Lei H, Li T, Li B, et al. Epstein-Barr virus from Burkitt Lymphoma biopsies from Africa and South America share novel LMP-1 promoter and gene variations. *Sci Rep*. Nov 23 2015;5:16706. doi:10.1038/srep16706

17. Palser AL, Grayson NE, White RE, et al. Genome diversity of Epstein-Barr virus from multiple tumor types and normal infection. *J Virol*. May 2015;89(10):5222-37. doi:10.1128/JVI.03614-14

18. Song KA, Yang SD, Hwang J, Kim JI, Kang MS. The full-length DNA sequence of Epstein Barr virus from a human gastric carcinoma cell line, SNU-719. *Virus Genes*. Dec 2015;51(3):329-37. doi:10.1007/s11262-015-1248-z

19. Kanda T, Furuse Y, Oshitani H, Kiyono T. Highly Efficient CRISPR/Cas9-Mediated Cloning and Functional Characterization of Gastric Cancer-Derived Epstein-Barr Virus Strains. *J Virol*. May 2016;90(9):4383-93. doi:10.1128/JVI.00060-16

20. Liu Y, W Y, Pan Y, Ji J, Lu Z, Y. K. Genome-wide analysis of Epstein-Barr virus (EBV) isolated from EBV-associated gastric carcinoma (EBVaGC). *Oncotarget*. 2016;7(4):4903-14.

21. Wang S, Xiong H, Yan S, Wu N, Lu Z. Identification and Characterization of Epstein-Barr Virus Genomes in Lung Carcinoma Biopsy Samples by Next-Generation Sequencing Technology. *Sci Rep*. May 18 2016;6:26156. doi:10.1038/srep26156

22. Xiao K, Yu Z, Li X, et al. Genome-wide Analysis of Epstein-Barr Virus (EBV) Integration and Strain in C666-1 and Raji Cells. *J Cancer*. 2016;7(2):214-24. doi:10.7150/jca.13150

23. Borozan I, Zapatka M, Frappier L, Ferretti V. Analysis of Epstein-Barr Virus Genomes and Expression Profiles in Gastric Adenocarcinoma. *J Virol*. Jan 15 2018;92(2)doi:10.1128/JVI.01239-17

24. Correia S, Bridges R, Wegner F, et al. Sequence Variation of Epstein-Barr Virus: Viral Types, Geography, Codon Usage, and Diseases. *J Virol*. 2018;92(22):e01132-18.

25. Hui KF, Chan TF, Yang W, et al. High risk Epstein-Barr virus variants characterized by distinct polymorphisms in the EBER locus are strongly associated with nasopharyngeal carcinoma. *Int J Cancer*. Jun 15 2019;144(12):3031-3042. doi:10.1002/ijc.32049

26. Okuno Y, Murata T, Sato Y, et al. Defective Epstein-Barr virus in chronic active infection and haematological malignancy. *Nat Microbiol*. Mar 2019;4(3):404-413. doi:10.1038/s41564-018-0334-0

27. Peng RJ, Han BW, Cai QQ, et al. Genomic and transcriptomic landscapes of Epstein-Barr virus in extranodal natural killer T-cell lymphoma. *Leukemia*. Jun 2019;33(6):1451-1462. doi:10.1038/s41375-018-0324-5

28. Xu M, Yao Y, Chen H, et al. Genome sequencing analysis identifies Epstein-Barr virus subtypes associated with high risk of nasopharyngeal carcinoma. *Nat Genet*. Jul 2019;51(7):1131-1136. doi:10.1038/s41588-019-0436-5

29. Wu YX, Zhang WL, Wang TM, et al. Genomic Landscapes of Epstein-Barr Virus in Pulmonary Lymphoepithelioma-Like Carcinoma. *J Virol*. 2022;96(4):e0169321.

30. Katoh K, Standley DM. MAFFT Multiple Sequence Alignment Software Version 7: Improvements in Performance and Usability. *Molecular Biology and Evolution*. 2013;30(4):772-780. doi:10.1093/molbev/mst010

31. Danecek P, Auton A, Abecasis G, et al. The variant call format and VCFtools. *Bioinformatics*. Aug 1 2011;27(15):2156-8. doi:10.1093/bioinformatics/btr330

32. Price AL, Patterson NJ, Plenge RM, Weinblatt ME, Shadick NA, Reich D. Principal components analysis corrects for stratification in genome-wide association studies. *Nat Genet*. 2006;38(8):904-9.

33. Alexander DH, Novembre J, Lange K. Fast model-based estimation of ancestry in unrelated individuals. *Genome Res*. Sep 2009;19(9):1655-64. doi:10.1101/gr.094052.109

34. Pickrell JK, Pritchard JK. Inference of population splits and mixtures from genome-wide allele frequency data. *PLoS Genet*. 2012;8(11):e1002967. doi:10.1371/journal.pgen.1002967

35. Gao Y, Zhang C, Yuan L, et al. PGG.Han: the Han Chinese genome database and analysis platform. *Nucleic Acids Res*. Jan 8 2020;48(D1):D971-D976. doi:10.1093/nar/gkz829

36. Chang CC, Chow CC, Tellier LC, Vattikuti S, Purcell SM, Lee JJ. Second-generation PLINK: rising to the challenge of larger and richer datasets. *Gigascience*. 2015;4:7. doi:10.1186/s13742-015-0047-8

37. Paradis E, Schliep K. ape 5.0: an environment for modern phylogenetics and evolutionary analyses in R. *Bioinformatics*. Feb 1 2019;35(3):526-528. doi:10.1093/bioinformatics/bty633

38. Revell LJ. phytools: an R package for phylogenetic comparative biology (and other things). *Methods in Ecology and Evolution*. 2012;3(2):217-223. doi:10.1111/j.2041-210X.2011.00169.x

39. Croucher NJ, Page AJ, Connor TR, et al. Rapid phylogenetic analysis of large samples of recombinant bacterial whole genome sequences using Gubbins. *Nucleic Acids Res*. Feb 18 2015;43(3):e15. doi:10.1093/nar/gku1196

40. Stamatakis A. RAxML version 8: a tool for phylogenetic analysis and post-analysis of large phylogenies. *Bioinformatics*. May 1 2014;30(9):1312-3. doi:10.1093/bioinformatics/btu033

41. Rice P, Longden I, Bleasby A. EMBOSS: the European Molecular Biology Open Software Suite. . *Trends Genet*. 2000;16(6):276-7.

42. Martin DP, Varsani A, Roumagnac P, et al. RDP5: a computer program for analyzing recombination in, and removing signals of recombination from, nucleotide sequence datasets. *Virus Evol*. Jan 2021;7(1):veaa087. doi:10.1093/ve/veaa087

43. Lee CC, Huang TT, Lee MS, et al. Survival rate in nasopharyngeal carcinoma improved by high caseload volume: a nationwide population-based study in Taiwan. *Radiat Oncol*. Aug 11 2011;6:92. doi:10.1186/1748-717X-6-92

44. Center NC. *CHINA CANCER REGISTRY ANNUAL REPORT 2020.* People's Medical Publishing House; 2022.

45. Bouckaert R, Vaughan TG, Barido-Sottani J, et al. BEAST 2.5: An advanced software platform for Bayesian evolutionary analysis. *PLoS Comput Biol*. Apr 2019;15(4):e1006650. doi:10.1371/journal.pcbi.1006650

46. Rambaut A DA, Xie D, Baele G, Suchard MA. Posterior summarisation in Bayesian phylogenetics using Tracer 1.7. *Systematic Biology*. 2018;syy32

47. Jensen TZT, Niemann J, Iversen KH, et al. A 5700 year-old human genome and oral microbiome from chewed birch pitch. *Nat Commun*. Dec 17 2019;10(1):5520. doi:10.1038/s41467-019-13549-9

48. Pfeifer B, Wittelsburger U, Ramos-Onsins SE, Lercher MJ. PopGenome: an efficient Swiss army knife for population genomic analyses in R. *Mol Biol Evol*. Jul 2014;31(7):1929-36. doi:10.1093/molbev/msu136

49. Paradis E. pegas: an R package for population genetics with an integrated-modular approach. *Bioinformatics*. Feb 1 2010;26(3):419-20. doi:10.1093/bioinformatics/btp696

50. Hudson RR. Generating samples under a Wright-Fisher neutral model of genetic variation. *Bioinformatics*. Feb 2002;18(2):337-8. doi:10.1093/bioinformatics/18.2.337

51. Rasmus N, Slatkin M. *An Introduction to Population Genetics*. Sinauer Associates.; 2013.

52. Bruzzi P, Green SB, Byar DP, Brinton LA, Schairer C. Estimating the population attributable risk for multiple risk factors using case-control data. *Am J Epidemiol*. Nov 1985;122(5):904-14. doi:10.1093/oxfordjournals.aje.a114174

53. Zhou X, Cao SM, Cai YL, et al. A comprehensive risk score for effective risk stratification and screening of nasopharyngeal carcinoma. *Nat Commun*. Aug 31 2021;12(1):5189. doi:10.1038/s41467-021-25402-z

54. Feng FT, Cui Q, Liu WS, et al. A single nucleotide polymorphism in the Epstein-Barr virus genome is strongly associated with a high risk of nasopharyngeal carcinoma. *Chin J Cancer*. Dec 16 2015;34(12):563-72. doi:10.1186/s40880-015-0073-z
